# Ultrafast Near‐Ideal Phase‐Change Memristive Physical Unclonable Functions Driven by Amorphous State Variations

**DOI:** 10.1002/advs.202204453

**Published:** 2022-11-13

**Authors:** Shao‐Xiang Go, Qiang Wang, Kian Guan Lim, Tae Hoon Lee, Natasa Bajalovic, Desmond K. Loke

**Affiliations:** ^1^ Department of Science Mathematics and Technology Singapore University of Technology and Design Singapore 487372 Singapore; ^2^ Department of Engineering University of Cambridge Trumpington Street Cambridge CB2 1PZ UK; ^3^ School of Materials Science and Engineering Kyungpook National University Daegu 41566 Republic of Korea

**Keywords:** amorphous materials, digital memory, security, function, simulations

## Abstract

There is an ever‐increasing demand for next‐generation devices that do not require passwords and are impervious to cloning. For traditional hardware security solutions in edge computing devices, inherent limitations are addressed by physical unclonable functions (PUF). However, realizing efficient roots of trust for resource constrained hardware remains extremely challenging, despite excellent demonstrations with conventional silicon circuits and archetypal oxide memristor‐based crossbars. An attractive, down‐scalable approach to design efficient cryptographic hardware is to harness memristive materials with a large‐degree‐of‐randomness in materials state variations, but this strategy is still not well understood. Here, the utilization of high‐degree‐of‐randomness amorphous (A) state variations associated with different operating conditions via thermal fluctuation effects is demonstrated, as well as an integrated framework for in memory computing and next generation security primitives, viz., APUF, for achieving secure key generation and device authentication. Near ideal uniformity and uniqueness without additional initial writing overheads in weak memristive A‐PUF is achieved. In‐memory computing empowers a strong exclusive OR (XOR‐) and‐repeat A PUF construction to avoid machine learning attacks, while rapid crystallization processes enable large‐sized‐key reconfigurability. These findings pave the way for achieving a broadly applicable security primitive for enhancing antipiracy of integrated systems and product authentication in supply chains.

## Graphical abstract

A strategy is developed for key generation and device authentication through the utilization of high‐degree‐of‐randomness amorphous (A) state variations concomitant with different operating conditions to address the challenge of realizing efficient roots of trust for resource constrained hardware. This approach enables excellent physical unclonable functions (PUF) performance in uniformity, uniqueness, writing overheads, and machine learning attack resilience.

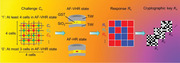

## 1 Introduction

Due to weak construction, reuse or data breaches, the problem with traditional passwords is that they are vulnerable.^[^ ^1^, ^2^ ^]^ When conventional passwords are on the security agenda, issues of theft and interception come to the fore in the world of supply‐chain product authentication.^[^ ^3^, ^4^ ^]^ Moreover, situations are not getting better when it comes to prototypical holograms or quick response (QR) codes, where imitation is rife.^[^ ^5^, ^6^ ^]^ Currently, the ever increasing reliance on connected network devices to manage large amount of confidential digital data and perform sensitive security tasks in everyday life is driven by emergence of the edge computing framework within Internet of Things (IoT) ecosystem.^[^ ^7^, ^8^, ^9^ ^]^ Edge‐computing device operations, based on the easy access to secure data such as credit card information, biometrics, authentication tokens for financial transactions and email passwords, making them attractive targets/gateways to terror cyber‐attacks, pose a large risk to information security.^[^ ^10^, ^11^, ^12^ ^]^ A need arises to develop hardware cryptographic solutions due to the inability of archetypal pure software‐based security to tackle the ever‐rising threats of cyberspace.^[^ ^13^, ^14^ ^]^ To store a secret key that is utilized by different cryptographic methodologies, typical hardware cryptographic approaches harness an array of non‐volatile memories.^[^ ^15^, ^16^ ^]^ According to the Kerckhoff's rule and Shannon's principles, the key is the major unknown variable that an attacker needs to recover or break a cryptographic system, so the security of a key is of vital importance.^[^ ^1^, ^17^ ^]^ However, due to the nonvolatile nature of prototypical digital memories, which retain information when the power is turned off, achieving multiple types of PUF for different applications is extremely challenging.^[^ ^19^, ^20^, ^21^ ^]^ Additionally, as copies of the key can be generated, it is difficult to bind the key to a targeted device.

The end users need to know the information of a product and be able to control that it is not a fake to mitigate the supply chain counterfeit problem.^[^ ^22^, ^23^ ^]^ A digital twin of each product where the information is stored and that users can access using a smart device is required.^[^ ^24^, ^25^ ^]^ Products need to have a unique identifier valid in both domains, that is, a connection between the physical and digital world, to create digital twins.^[^ ^26^, ^27^ ^]^ A physical identifier that is unique and cannot be copied is required to realize the intrinsic potential of generating digital twins of individual products that link physical and digital identities.

With the ability to create unique secret bit strings rapidly, physical unclonable functions (PUF) are promising candidates for next‐generation cryptographic methodologies.^[^ ^15^, ^28^ ^]^ PUF, based on stochastic variations in operating conditions for a component, exhibiting unique characters in physical signatures, makes it extremely difficult to duplicate a key.^[^ ^29^, ^30^ ^]^ A secret challenge‐response pair (CRP) space that is known only to a verifier results from modeling PUF as a mathematical function that maps the inputs/challenges (*c*) to outputs/responses (*r*), viz., (*r* = *f*(*c*)).^[^ ^31^, ^32^ ^]^ Depending on whether the number of CRPs are linearly or supra‐linearly related to the number of physical random elements in the circuit, the PUF is grouped as weak PUF or strong PUF.^[^ ^33^, ^34^ ^]^ Strong PUF are harnessed for applications such as authentication which requires the creation of multiple keys, whereas weak PUF are utilized for hardware identification.^[^ ^33^, ^35^ ^]^ Complementary‐metal‐oxide semiconductor (CMOS) technologies, for example, alternate frequencies of electronic oscillators (ring‐oscillator PUF), randomness of initial state of static‐random‐access memories (SRAM PUF) and different gate delays in multiplexers (arbiter PUF) are used in traditional PUF designs.^[^ ^1^, ^33^ ^]^ Recently, computer designers are able to harness new state variables such as impedance to represent information thanks to the emergence of conventional nanotechnologies and prototypical semiconducting materials systems and device designs.^[^ ^36^, ^37^ However, these technologies may generate new approaches for attack and result in unacceptable current defense, although they offer unique and ultra‐efficient circuit opportunities for PUF design.^[^ ^33^, ^38^, ^39^ ^]^ Thus, evaluating strong “roots of trust” in the case of new nanoscale device designs is urgently needed.

By powering new non‐von Neumann concepts such as neuromorphic hardware and in‐memory computing, memristor is a promising technology that is envisaged to push the future of computing beyond Moore's law.^[^ ^40^, ^41^ ^]^ Memristor functions, based on the utilization of randomness present within variations in material states, demonstrating rapid extraction of digital bits, are able to render the reading of a key directly particularly challenging.^[^ ^42^, ^43^ ^]^ Unique PUF designs that utilize the intrinsic stochasticity in physical signatures as sources of randomness are demonstrated in recent memristor studies.^[^ ^44^, ^45^, ^46^ ^]^ However, traditional memristive‐based PUF may rely on extended programming times, non‐ideal signatures and additional initial writing overheads (i.e., devices are switched before key generation) to achieve viable operational metrics.

**Figure 1:**
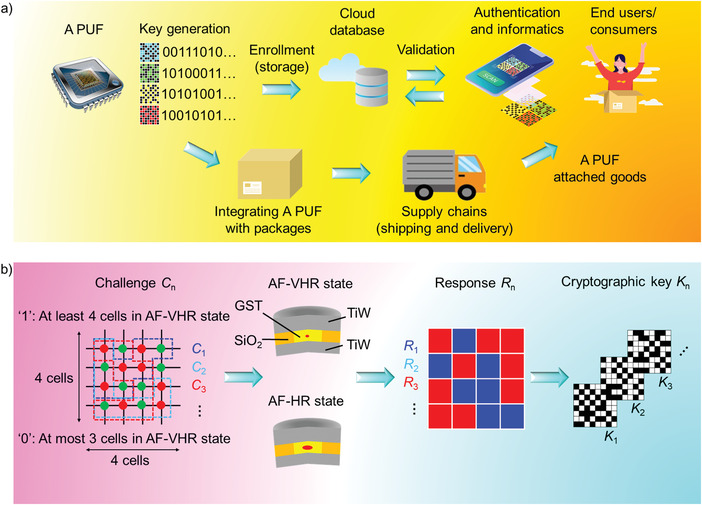
Amorphous‐based PUF (A PUF). a) Schematic illustration of the product authentication concept. The goods/packages can be attached with the A PUF by the original manufacturer. A unique PUF ID will be generated for each product. By accessing enrolled keys in a secure cloud database, end users, for example, consumers and other users, can validate the product and certify the source. Other useful information such as the product expiration date, supply chain path and other information relevant to the targeted product can also be stored in the A PUF. b) New types of PUF can be designed using phase‐change materials (PCM), based on the reversible switching between the melt‐quenched amorphous state and fully crystallized state of a chalcogenide layer, along with sensing different as‐deposited amorphous states, showing a large contrast in electrical and optical signatures. In this work, unique cryptographic keys are generated by the large degree of randomness of the as‐fabricated/melt‐quenched amorphous state of an ensemble of A PUF cells. The A PUF is implemented based on the design flow: i) the cell resistance is read using a differential readout strategy, ii) high stochasticity of the as‐fabricated or melt‐quenched state is enabled by different fabrication/cycling conditions, iii) the stochasticity is reflected by the difference in the resistance of cells, and iv) the difference in the as‐fabricated/melt quenched state is utilized to generate cryptographic keys.

Herein, we show that corresponding to different operating conditions, we are able to harness high‐degree‐of‐randomness amorphous (A) state variations, and also for achieving secure key generation and device authentication (**Figure** **1**), we demonstrate the development of an integrated framework for in memory computing and strong hardware security primitive, that is, A PUF. The approach is based on the idea of utilizing stochastic variations associated with fabrication/switching processes via thermal fluctuations (variation in temperature distributions of active layer), which enables the generation of A state variations with a large degree of randomness. Rapid reconfigurable A‐PUF with near ideal reconfigurability is achieved, as well as weak A PUF with near‐optimal uniqueness and uniformity without additional initial writing overheads. We utilize concepts of eXclusiveOR (XOR‐) and‐repeat operation to generate a strong A‐PUF strategy, in order to combat machine learning algorithms that builds models of randomness by CRP eavesdropping.

## 2 Results

An unique opportunity to design highly secure PUF with a large degree of randomness using novel memristive systems such as phase‐change materials (PCM) exists but is not well understood. Phase‐change (PC) system operations, based on the reversible switching between the crystalline and melt‐quenched amorphous states of a chalcogenide layer, as well as sensing different as‐deposited amorphous states, showing large contrast in electrical conductivity and optical reflectivity, are able to generate systems with high degrees of freedom.^[^ ^47^, ^48^ ^]^ To design highly secure PUF, these phenomena can be harnessed as novel sources of entropy, but are not well documented. The possibility of high performance hardware opens up as a result of the ability of these materials systems to switch on several ten nanosecond timescales.^[^ ^49^, ^50^ ^]^ High performing hardware‐based security systems are of vital importance as high performance hardware are poised to be integrated in mass‐manufactured products and portable electronics.^[^ ^7^, ^51^ ^]^ Innovations in data intensive/data readily‐accessible applications such as the IoT and consumer electronics are expected to be propelled by these high performance hardware built on a new generation of advanced materials systems.^[^ ^52^, ^53^ ^]^ However, the hardware security aspects in these frameworks is not well defined, despite remarkable progress in the realization of rapid and scalable hardware with memristive components.

High degree of entropy, viz., the existence of large degrees of randomness in electrical signatures that would act as a black box to not only the attacker but also to the original manufacturer, should be demonstrated in an ideal PUF materials system. An ideal candidate platform for next‐generation PUF is the as‐deposited state of PCM.^[^ ^54^, ^55^ ^]^ Experiments have demonstrated that the as‐deposited state exhibits a high degree of entropy.^[^ ^54^, ^56^ ^]^ The vapor deposition process induces rapid quenching of atoms from the vapor phase, so varied deposition conditions for different sample batches may generate various populations of subcritical nuclei in as‐deposited states;^[^ ^54^, ^57^ ^]^ in terms of the language of kinetic theory of nucleation, this may correspond to the different cluster‐size distributions in the matrix, which will be discussed in detail later. Moreover, recent studies have disclosed that PCM may not fully fill nanosized active regions initially due to void and overhang generated after deposition.^[^ ^58^, ^59^, ^60^ ^]^ Thus, in this work, the interest is in the utilization of PC systems in different as‐fabricated states/conditions as the entropy source for PUF design.

**Figure 2:**
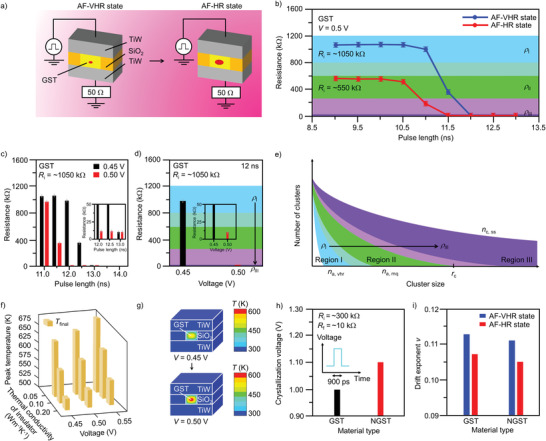
The A state variation upon electrical stimuli. a) Schematic illustration of the cell structure for different material states. b) Schematic Resistance‐pulse Length (RL) diagram. The RL diagram can be classified into three distinct regions *ρ*_I_–*ρ*_III_. The blue region (*ρ*_I_) represents the as‐fabricated very high resistance (AF‐VHR) state, while the as‐fabricated high resistance (AF‐HR) state/melt quenched high resistance state is described by the green region (*ρ*_II_). The purple region (*ρ*_III_) denotes the crystallized low resistance state. The cyan and violet regions define the boundaries between *ρ*_I_ and *ρ*_II_, and between *ρ*_II_ and *ρ*_III_, respectively. The blue and green regions disclose the range of data obtained from experiments performed on three different batches of cells in the AF‐VHR state and AF‐HR state, respectively. The range of values from experiments carried out on three different cells in a batch is denoted by error bars. c,d) Dependence of the resistance on the c) pulse length for different stimulus voltages, and d) stimulus voltage for the pulse length of 12 ns. The error bars represents the range of values from experiments performed on three different cells in a batch. e) Schematic description of the evolution of cluster‐size distributions after applying stimuli corresponding to different regions in the RL diagram. f) Variation of peak temperature for different stimulus voltages and thermal conductivities of insulating layer. g) Thermal distributions of the cell upon the application of different stimuli amplitudes. h) Materials type dependent crystallization voltage of the cell. i) Drift exponent variation for different materials types and with different material states.

We demonstrate the temporal evolution of amorphous (A) states upon the application of electrical stimuli. **Figure** **2**a shows the schematic representation of the PC cell. The cell has a pore‐like structure composed of a 20 nm‐thick GeSbTe‐based layer, which was sandwiched between 300 nm‐thick TiW top and bottom electrodes. The GeSbTe‐based layer was confined in the 30 nm‐wide pores generated by 20 nm‐thick SiO_2_ insulation layer. The silica insulator provides electrical and thermal insulation, while the electrodes were utilized to connect the test structures to the external circuitry for electrical testing. Three different materials state types, each having a different resistance range, were utilized. Figure 2a,b show that each state represents a different region in the Resistance‐stimulus Length (RL) diagram: i) The very‐high‐resistance (VHR) amorphous state is defined as region I; ii) the high‐resistance (HR) amorphous state as region II; and iii) the low‐resistance (LR) crystallized state as region III. Varying stimulus length results in a different influence on subsequent states in Figure 2b. After applying a long stimulus (i.e., ≈11 ns), the onset of switching from the as fabricated VHR state to the LR state emanates. On the other hand, the onset of switching from the as‐fabricated HR state to the LR state occurs upon the application of a shorter stimulus (viz., ≈10.5 ns). Thus, the transition from region I to region III is associated with an extended stimulus time, whereas a short stimulus duration corresponds to the case for region‐II‐to‐region‐III transition. An interesting observation is that, when a stronger stimulus is applied (from 0.45 to 0.5 V), the onset of switching from the VHR state to the LR state occurs with a shorter stimulus (from 12 ns to 11 ns) (Figure 2c), corresponding to a decreased stimulus time for the transition from region I to region III. Moreover, the change in the transition type from the active layer that remains in the region I to the active layer that undergoes the region‐I‐to‐region‐III transition is described by the variation in process from negligible switching to the switching from the VHR state to the LR state after a strong stimulus is administered (Figure 2d).

Cluster size distributions that form the basis of kinetic theory of nucleation can represent A states, and their evolution may describe the change in the resistance state upon specified electrical stimuli. The degree of the medium range order in the disordered network structure that fluctuates locally and temporally upon excitations may depict A states on microscopic scale.^[^ ^61^, ^62^ ^]^ In response to a temperature change, the cluster population of the VHR amorphous state *n*_a,vhr_ evolves gradually to the steady state distribution *n*_c,ss_ at an elevated temperature in a finite time period, as shown by computations based on the kinetic theory of nucleation (Figure 2e).^[^ ^63^, ^64^ ^]^ The thermally activated process during the growth of cluster, which involves atomic attachment/detachment through thermal fluctuations, induces this time dependence.^[^ ^47^, ^65^ ^]^ Upon the application of an electrical stimulus via Joule heating, the cell temperature increases in PC systems. The cluster distribution starts to evolve from the cluster population at a specified time *n*(*t*) after the injection of an electrical stimulus. When the size of largest clusters in the *n*(*t*) lies further away from the *n*_a,vhr_, the expectation time for clusters to grow in small populations shortens. As disclosed in Figure 2b, the stimulus time is thus a function of the *n*(*t*) at the time of the administration of an electrical stimulus, which explains the mechanism of a shorter stimulus with the change in the process from the VHR‐state‐to‐LR‐state switching to the HR‐state‐to‐LR‐crystallized‐state switching. Furthermore, the broad cluster distribution takes into account the influence of the spatial‐temperature distribution that is expected to rise in PC cells after the application of an stimulus, as described in Figure 2e.

From a theoretical modeling point of view, switching characters of the A state is the result of temperature changes in active layer. An electrothermal simulation of thermal distributions in cell models by simplifying the problem to the application of electrical stimulus to generate Joule heating also predicts that the different switching processes induced are the result of temperature variations in active layer. The models disclose an increased peak temperature with an increasing stimulus amplitude (Figure 2f,g, Figure S1, Supporting Information). Therefore, the active layer that remains in region I, showing negligible switching, can be represented by a model with a decreased peak temperature generated in the active layer, whereas an increasing peak temperature corresponds to the case where the transition from region I to the region III occurs (Figure 2d). This demonstrates the tunability of A state switching signatures by varying temperature conditions.

Up to this point, the pristine GeSbTe has been utilized to induce A states. We developed a method by which we can generate the A state by harnessing GeSbTe doped with nitrogen, which suppresses the crystallization process,^[^ ^66^, ^67^ ^]^ without harnessing bare GeSbTe. This requires the utilization of GeSbTe doped with 3 at% nitrogen (Figure 2h). After setting the initial state of the cell to the reset state, the nitrogen‐doped GeSbTe shows a higher stimulus voltage for crystallization compared to that for pure GeSbTe (Figure 2h), upon the application of different bias voltages to crystallize the active layer (see Figure S2, Supporting Information). Figure 2i, Figure S3, Supporting Information shows the drift exponent of A states for different active layers. The change in material characters induced by structural relaxation results in a large drift exponent *v* in pure active layer (*v* is calculated using $R=R_{0}{\left(\frac{t}{t_{0}}\right)}^{v}$, where *R* is the resistance, *t* denotes the time, *t*_0_ is the arbitrarily‐chosen‐zero time and *R*_0_ describes the resistance at *t* = *t*_0_).^[^ ^68^ ^]^ When the active layer is doped with nitrogen, the formation of strong Ge—N bonds increases the rigidity of the network, which leads to an enhanced thermal stability of material signatures and smaller drift exponent. A lower drift exponent of A states is exhibited by the nitrogen doped GeSbTe than that for pure GeSbTe (Figure 2i), which indicates that the stability of the material state can be facilitated via materials engineering.

The 10‐year data retention of A states for different active layers is further shown in Figure S4, Supporting Information. Experiments have demonstrated rapid crystallization kinetics at elevated temperature for GeSbTe doped with nitrogen, and at the same time, maintained excellent stability of the reset state at room temperature.^[^ ^66^, ^69^ ^]^ The 10‐year data‐retention ability of the VHR state of the nitrogen‐doped GeSbTe (≈374 K) is better than that of pristine GeSbTe (362 K). These findings indicate that crystallization is suppressed in the A GeSbTe doped with nitrogen near the room temperature, relative to that at elevated temperatures in the set operation, despite the enhanced driving force for crystallization.

**Figure 3:**
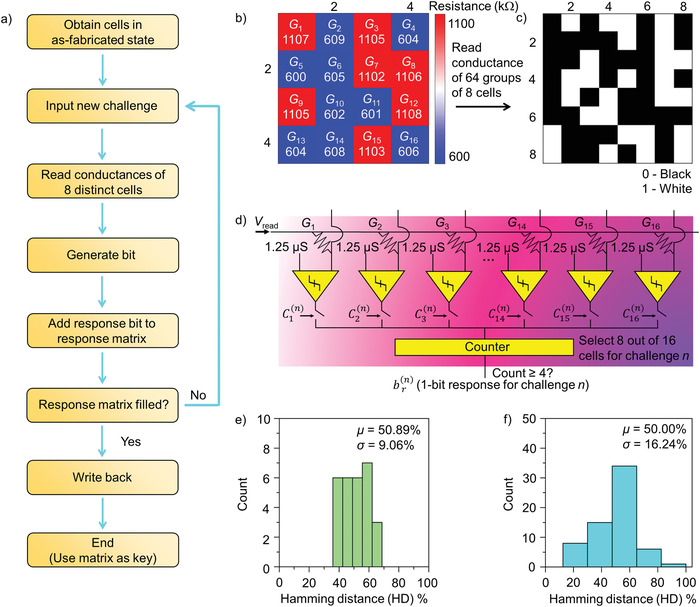
Weak A PUF. a) Flowchart illustrating the A PUF bit generation. b) Heat map of the resistance of cells with VHR/HR as‐fabricated states exhibiting a high degree of randomness. The cell resistance was measured using a reading voltage of 0.2 V. c) Digital maps upon conversion of response to binary bit. d) Circuit architecture of A PUF cells, with differential sensing via a comparator. e,f) Inter‐hamming distance (HD) histograms for the e) uniqueness (UQ) and f) uniformity (UF) harnessing eight groups of 64‐bit digitized keys.

Finally, we investigate the utilization of these A states as anti‐counterfeiting tags (we call them A tags). The A‐tags are material states, while the A key, which is represented by the digital map, is generated through transforming a random set of resistance values for an ensemble of cells with as‐fabricated states to binary digits. By applying a weak stimuli, the reading of A tags can be achieved. The first key generation methodology based on direct analyses was designed (the term weak PUF is utilized for this case) (**Figure** **3**a). Figure 3b shows the reading of A tags by measuring the resistance of cells obtained from a 4 × 4 cell array. Cumulative probability distribution plots of the VHR and HR for the as‐fabricated cell disclose small variations among the cells and arrays for both VHR and HR (Figure S5, Supporting Information). Additionally, based on the cell resistance/conductance that generate a response “0” or “1”, the A key is created in Figure 3c. Figure 3d shows the circuit to generate the response bit “0”/“1” by comparing the conductance of the cell *G*_n_ with the reference conductance *G*_ref,1_ (1.25 µS), and then performing counter operations. Further details are provided in the Supporting Information (see weak PUF section). The conductance values (viz., *G*_1_ to *G*_16_) were calculated utilizing the data in Figure 3b. The response “1” results if cells exhibit a conductance below the *G*_ref,1_ (i.e., cells in VHR state are observed) for at least 4 out of 8 selected cells, whereas in the case of cells that disclose a conductance above *G*_ref,1_ (viz., we attain cells in HR state) for at least 5 out of 8 selected cells, the response “0” is induced. Thus, if at least 4 out of 8 cells remain in region I, the response “1” occurs, while the response “0” corresponds to the case of at least 5 out 8 cells that stay in region II. A 64‐bit key (each bit corresponding to a response) was generated based on a group of 64 different challenges, where each challenge corresponds to the selection of 8 distinct cells out of 16 cells for conductance computation.

To calculate the uniqueness (UQ) of A state variations, viz., the differences in responses to the same set of challenges for different instances (i.e., altered positions of cells) of a PUF design, we computed the hamming distance (HD) distribution for the UQ of cells (Figure 3e). Different instances of PUF with a high number of unique response sets disclose a mean value of ≈50%, and the mean value diverges away from 50% for different instances of PUF with a low number of unique response sets.^[^ ^70^, ^71^ ^]^ We obtained a mean value of 50.89% with a standard deviation of 9.06%, indicating that a large number of unique response sets can be induced through the generation of a specific distribution of cells in region I/II. Moreover, we calculated the HD distribution for uniformity (UF) to estimate the UF of A state variations, that is, the number of times the output is “1” out of *m* observations (Figure 3f). A large degree of bias results for the case where the mean value is further away from 50% (the response can be guessed easily), while the mean value becomes close to 50% if the degree of bias is small (it is difficult to guess the response).^[^ ^70^, ^71^ ^]^ The generation of a specified distribution of cells in region I/II results in low‐degree‐of‐bias response sets, since a mean value of 50.00% with a standard deviation of 16.24% is attained.

**Figure 4:**
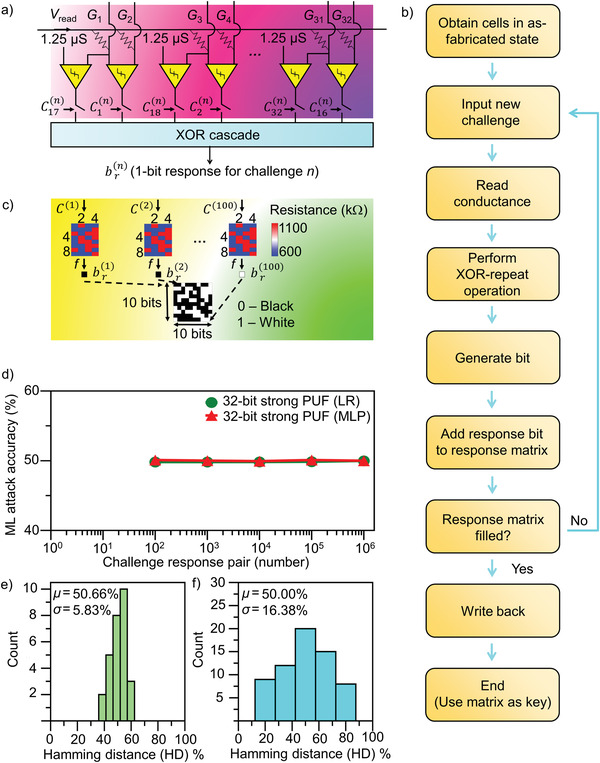
Strong A PUF resilient to machine learning attacks. a) Strong PUF architecture with a final 1‐bit response. Challenge consists of 32 bits, where each bit determines whether the switch is turned on to generate intermediate bits for XOR cascade. b) Strong‐PUF flowchart showing how the strong PUF is achieved. c) Conceptual diagram showing the repeated PUF configuration exhibited as an unrolled cascade of PUF without repeating processes. After each repeating step using different challenges, the process is stopped to enable the output bit *R*(*n*) to be read and inserted into the response matrix until 100 bits are generated to form the 10 × 10 key. d) Machine learning (ML) accuracy as a function of CRPs utilized for training and with two different ML models, logistic regression (LR) and multilayer perception (MLP). e,f) Inter‐HD histograms for the e) UQ and f) UF.

A problem of the A‐state PUF strategy above is that it may be susceptible to machine learning (ML) attacks. If we consider the weak PUF for the generation of CRPs, the response is generated through calculating the number of cells that exhibit a conductance above the *G*_ref,1_ out of an arbitrary group of 8 cells. As shown in Figure S6, Supporting Information, a variety of machine learners such as the random forest (RF) and logistic regression (LR) algorithms can be utilized to model the CRP relationship in this case and achieve an accuracy of ≈75% and 92.5% for 100 CRPs harnessed for training, respectively. In the Supporting Information (see “security of weak PUF” section), further details are provided. We tackle this issue by utilizing the XOR‐and‐repeat operation to render the CRP relationship more obscure for machine learners to compute^[^ ^72^, ^73^, ^74^ ^]^ (we define it as strong PUF) (**Figure** **4**a,b). The idea involves comparing between the conductance of a pair of cells *G*_n_, *G*_n + 1_, as well as the *G*_n_ and *G*_ref,1_ (i.e., 1.25 µS), and then performing the XOR operation to generate response bit “0”/“1”. The cell conductance *G*_1_ to *G*_32_ of the circuit was calculated using resistance values achieved from a 8 × 4 cell array (Figure S7, Supporting Information). In Figure 4c, based on the cell resistance/conductance value that creates a response bit “0” or “1”, the A key is generated. Further details are provided in the Supporting Information (see “strong PUF” section). The response bit created depends on the number of instances two events occur. In event 1, the challenge bit is “1”, and the *G*_n_ and *G*_n + 1_ are different. In event 2, the challenge bit is “1” and the *G*_n_ is smaller than *G*_ref,1_. As a result, event 1 corresponds to one cell in region I and the other cell in region II, while the case of the cell that remains in region I is represented by event 2. The response bit is “1” if the total number of times these events occur is odd, whereas in the case that none of these events emanate/the total number of times these events take place is even, response bit “0” results. This process is repeated 100 times to obtain 100 response bits. The key is then generated after filling the 100‐bit/10 × 10 response matrix.

Testing ML attacks on these A‐state responses with different algorithms, viz., LR and multi‐layered perception (MLP), generate a maximum attack accuracy of ≈50% (close to the 50% accuracy of a wild guess, away from the 100% accuracy for the case of easy guessing) (Figure 4d). This indicates that the generated distribution of cells in region I/II induces responses with a strong resistance against ML attacks. Moreover, UQ distribution calculations show that the PUF reveals a mean value of 50.6% with a standard deviation of 5.8% (Figure 4e), which indicates that, through generating a specified distribution of cells in region I/II, a large number of unique response sets results. Additionally, low‐degree‐of‐bias response sets are induced by the generation of specific region I/II cell distributions, as the mean value of 50.0% with a standard deviation of 16.3% for the UF distribution of A‐PUF is obtained (Figure 4f). Additionally, the cells passed all 15 National Institute of Standards and Technology (NIST) tests (see Table S1, Supporting Information), indicating that the quality of random responses resulting from the creation of a targeted distribution of cells in region I/II is high.

To support ownership change, change in privileges or preventing information leakage due to overuse, the reconfiguration of A states is required. Previous PUF keys inferred and documented by an attacker becomes useless when the trustworthy party reconfigure the key through physical reconfigurability.^[^ ^75^, ^76^ ^]^ We harness the cycle‐to‐cycle variability of PCM^[^ ^77^, ^78^ ^]^ to switch cells into new reset states to facilitate the freshness of cryptographic key. The reset/melt‐quenched state may comprise different amounts and sizes of subcritical nuclei if different cooling rates are generated, thus exhibiting a high degree of randomness, as disclosed in recent studies (albeit, for this work, with a smaller contrast in electrical conductance).^[^ ^47^, ^79^ ^]^ The cells were switched from the as‐fabricated VHR/HR state to the LR state, and then reversible switching between the set state and the reset state was performed (Figure S8a, Supporting Information). We obtain a new group of random resistances after every switching to the reset state due to cycle to cycle variations. The marked difference in the resistance distribution before and after reconfiguration is shown in Figure S8b, Supporting Information. Figure S8c, Supporting Information, exhibits the histogram of reconfiguration HD. The mean value of the HD distribution for reconfiguration is close to 50% if the degree of independence between reconfigured keys is high, whereas a low degree of independence between reconfigured keys results in a mean value that diverge away from 50%. A mean value of the reconfiguration HD distribution of ≈51.56% was achieved, indicating that the transition from region III to region II induces, between reconfigured keys, a high degree of independence. Furthermore, the cross correlation between reconfigured keys reveals a mean value of 0.17, as well as small values, indicating a large extent of independence between reconfigured keys (Figure S8d, Supporting Information). The write back technique further helps in alleviating reading errors due to other perturbations such as noisy conditions/spontaneous crystallization (Supporting Information, see “write back of weak/strong/reconfigured PUF” section).

The performance of cells for A state reconfiguration is high. The crystallization time (time needed to switch from the melt‐quenched amorphous state to the fully crystallized state) is longer than the amorphization time (the fully‐crystallized‐state‐to‐melt‐quenched‐amorphous‐state duration), which limits overall cell performance.^[^ ^80^, ^81^ ^]^ The crystallization rate, heating‐rate, time and temperature determines the crystal fraction of active layer.^[^ ^82^ ^]^ It is possible to control the crystal fraction and cell resistance by altering the amplitude and length of crystallization stimuli, since the crystal fraction controls cell‐resistance. The stimulus duration should be long enough to completely crystallize the active region for achieving the smallest resistance. The cells disclose a shorter crystallization stimulus length with an increase in bias amplitude (see Figure S9, Supporting Information). Moreover, the shortest crystallization‐stimulus length achieved is 900 ps. As a result, excellent performance is shown by the cells.

A small energy consumption for A‐state reconfiguration is also achieved. When a switching from the LR fully‐crystallized state to the HR melt‐quenched amorphous state is considered, the energy consumption is larger than that from the HR melt quenched state to the LR fully crystallized state due to the large resetting current required for PC cells. In this work, the largest reset current and energy is ≈0.6 mA and 1.4 pJ, respectively (*E* = *V* × *I* × *t* = 3.5 V × 0.6 mA × 700 ps, where *E* defines the reset energy, *V* is the stimulus amplitude, *I* represents the current and *t* is the pulse length). Thus, the cells exhibit small energy consumption.

Notably, in this work, the experiments and theoretical studies demonstrate the development of an integrated A state variation‐type PCM framework for in memory computing and next generation security primitive for realizing secure key generation and device authentication, for example, the group of weak PUF and strong PUF driven by PCM‐based cell‐to‐cell as‐fabricated A‐state variations, which has not been demonstrated before, as well as the utility of large‐degree‐of‐randomness A state variations in a PCM concomitant with different operating conditions through thermal fluctuation phenomena, that is, obtaining a previously unreported deeper level of insights into the VHR‐state‐to‐LR‐state switching via the cluster size distribution analysis and electrothermal simulations. Moreover, a previously undocumented near‐ideal uniformity and uniqueness without needing additional initial writing overheads in weak PUF via integrated as fabricated A‐state variation‐based PCM platform was achieved, along with a rapid, near‐ideal reconfigurability to refresh A keys enabled by the hybrid PCM A‐state variation‐archetype framework if necessary, which has not been achieved to date, in addition to a previously unseen PCM‐powered XOR‐and‐repeat scheme of response generation in strong PUF that enhances the security of memory cells against ML attacks.

## 3 Discussion

Applications such as hardware security primitives for secure key generation and device authentication are challenging for traditional memristive devices because of several requirements: i) Near‐ideal UQ/UF, ii) unclonable and secure (resistant to invasive attack), iii) reconfigurable, and iv) compact. Currently, a limited number of memristive devices fulfill all the requirements listed above. The examples shown in this work indicate that the current state of the PC cell may achieve most of these requirements with reasonable performance in relation to conventional memristive devices. The key improvement in PC cells to enable these applications is the enhanced UQ of the PUF, achieved by the utilization of unique double as‐fabricated A states, which have not been demonstrated before. The PC cell shows weak PUF with a mean value of HD distribution for UQ of ≈50.8%, which is ≈2.2% closer to the ideal value of 50% compared to the average of 47% for existing PUF using memristive‐based devices (Figure S10, Supporting Information). Moreover, the PC cell shows weak PUF with a mean value for the HD distribution for UF of ≈50.0%, which is ≈0.3% nearer to the 50% ideal value than the average of 49.63% for current PUF via memristive‐based devices (Figure S11, Supporting Information). Furthermore, the PC cell demonstrates strong PUF that pass all 15 NIST tests, which is ≈66% higher than the average of 9 tests obtained for current PUF using memristive‐based devices (Figure S12, Supporting Information). As a result, this makes it extremely difficult to decipher or infer the content stored in the hardware for improving storage security.

Another key advantage of the PC cell is the ML attack resilience performance. For instance, the PC cell demonstrates strong PUF with a maximum attack accuracy of ≈50%, which is ≈4.2% nearer to the 50% accuracy of random guessing than the average of 54.25% for state‐of‐the‐art PUF based on memristive devices (Figure S13, Supporting Information). Thus, this enables an unclonable and secure PUF for improving resistance to invasive attacks. The integration of the PC cell and PUF in the same system also reduces the attack surface (the exposure in terms of additional wires and pins that can be attacked by a hacker if the PUF is separated from the system), which further enhances resilience to ML attack.

The PUF demonstrated by PC cells has an additional advantage of reconfigurability. The mean value of HD distribution for reconfiguration discloses a value of 51.56%, which is ≈3.1% closer to the ideal value of 50% compared to the average of 45.26% for current PUF based on memristive devices (Figure S14, Supporting Information). Thus, this enables the creation of a different set of keys than what was generated before for achieving environmentally strong PUF.

The ability to integrate the PC cell and PUF in the same system confer a further advantage over traditional von Neumann hardware, which have separate storage and processing systems, by enabling a miniaturized hardware to carry out the same number of operations for facilitating device downscaling. Currently, a large time gap exists between traditional volatile memory technologies and typical nonvolatile memory technologies.^[^ ^71^, ^83^ ^]^ Conventional volatile memory technologies (viz., prototypical dynamic random‐access memory) have fast access times (nanosecond timescale), but the data is lost when the power supply is turned off (power needs to be turned on to retain data). Prototypical nonvolatile memory technologies (e.g., traditional flash memory) do not lose the data after the power is turned off, but the accessing speeds (in the order of milliseconds) are slower than that of volatile memory technologies. A new PUF‐in‐memory technology that possess a fast‐writing speed and does not lose the data after the power is turned off is required for bridging the large time gap between archetypal volatile memory technologies and typical nonvolatile memory technologies. In this work, the PC cell exhibits PUF with a crystallization stimulus length of 900 ps, which is approximately an order of magnitude shorter than the average of 50 ns for existing PUF using memristive devices (Figure S15, Supporting Information). This enables the utilization of downsized hardware for improving device miniaturization.

## 4 Conclusion

This near‐ideal uniqueness, as well as near perfect uniformity, and also without additional initial writing overheads in weak PUF are achieved in association with different operating conditions via thermal fluctuations that enables high‐degree‐of‐randomness A‐state variation utility. To enable reconfigurability of PUF keys with 51.56% inter‐HD between the reconfigured keys, we harness the rapid switching process of A PUF. Finally, to protect the PUF against modeling attacks, we demonstrate a strong A PUF harnessing in‐memory computing features and utilize XOR‐and‐repeat PUF construction strategies. In principle, this approach is applicable to a wide range of A materials and cell ensemble sizes, so that an appropriate combination of A states and computing schemes opens opportunities for optimizing A PUF performance.

## 5 Experimental Section

### Cell Fabrication and Materials Characterization

The PC cell was fabricated utilizing an integrated conventional lithography and nanopatterning technique, according to the previous fabrication protocol.^[^ ^84^, ^85^ ^]^ Each patterning step consisted using a 365 nm photolithography (Cannon) or electron‐beam lithography (JEOL), followed by the materials deposition and lift‐off process. All of the materials were deposited utilizing composite targets in a DC magnetron sputtering system (Blazers Cube). Nitrogen‐doped Ge_2_Sb_2_Te_5_ (NGST) films were deposited by sputtering from a composite GST target and concurrently in flowing nitrogen gas at a constant N_2_/Ar flow rate of 0.2. The NGST films were characterized using X‐ray photoelectron spectroscopy (XPS), which showed that the nitrogen concentration in the film is ≈3 at%. A 4ʺ Si wafer with a 1 µm‐thick SiO_2_ layer was utilized as the starting configuration, on which a 300 nm‐thick TiW bottom electrode was deposited and patterned. An insulating layer, consisting a 20 nm‐thick layer of SiO_2_, was deposited and etched to form pores with diameters of ≈30 nm. The openings were filled with a 20 nm‐thick layer of GST/NGST for forming the active regions. Finally, a 300 nm‐thick TiW top electrode was deposited to complete the structure.

### Cell Structure

The PC cell was deposited on a SiO_2_‐on‐Si subtrate, based on the previous cell structure.^[^ ^84^ ^]^ The cell has a pore‐like structure consisting a 20 nm‐thick GST/NGST layer, which was sandwiched between 300 nm‐thick top and bottom TiW electrodes. The GST or NGST was confined in the 30 nm‐wide pores formed by 20 nm‐thick SiO_2_ insulation layer. The electrodes were used to connect the test structures to the external circuitry for electrical testing, while the silica insulator provides electrical and thermal insulation.

### Electrical Characterization

The PC cell was characterized using a custom‐built electrical characterization system consisting of a picosecond (Picoseconds Pulse Lab) or nanosecond (Tektronix) pulse generator, a digital oscilloscope (Agilent Technologies), and a probe station, based on the previous testing protocol.^[^ ^85^, ^86^ ^]^ The nanosecond pulse generator had the specifications of pulse duration varying between 5–900 ns, a rise time of <3 ns, and a maximum amplitude of 5.0 V, and the picosecond pulse generator had the specifications of pulse durations ranging from 100 ps to 10 ns, rise time of 65 ps, and a maximum amplitude of 7.5 V. The cell was connected to the pulse generator and oscilloscope through low‐capacitance cables (≈0.2–3 pF) and a load resistor *R*_1_ = 50 Ω. The upper limit of the time constant of the resistance‐capacitance circuit was estimated to be several 10 ps. The full width at half maximum (FWHM) time duration of the pulse was measured before the signal passes through the cell and this was utilized to characterize the times of switching in the cells. This work had previously examined and disclosed the waveform of the voltage pulses obtained before the signal passes through the cell and after the signal passes through the cell. As in those studies, the waveform of the pulse obtained before the signal passes through the cell also reflected the exact voltage pulse that was injected to the cell, taking into account the capacitance or inductance of the probe circuitry and connectors. The FWHMs of the waveforms obtained before the signal passes through the cell and after the signal passes through the cell were almost the same. Moreover, as the pulse obtained after the signal passes through the cell had moved through the structure, the duration of the pulse experienced by the cell was almost identical to that of the pulse entering the cell. Furthermore, a comparison of the shapes of the pulses obtained before the signal passes through the cell and after the signal passes through the cell also showed that parasitic‐capactitance effects in the circuit or structure were negligible. This work had utilized one of the conventional structures harnessed by many other research groups.^[^ ^87^ ^]^ The voltage‐pulse duration needed to switch the large cells was several tens of nanoseconds (depending on the voltage applied), which was about the same as those achieved by other research groups.^[^ ^88^ ^]^ The duration and height of the voltage pulses were varied from several 100 ps to several 10 ns and from 0 to 7 V, respectively. To ensure good functionality, the cells were switched reversibly more than 100× between the low‐resistance level of ≈10 kΩ and the high resistance level of ≈300 kΩ before the experiemental study. The occurrence of crystallization was determined by the change of the resistance level of the cell. The resistance change of the cell with small pore size was of “sudden drop” type and the cell with large pore size showed similar behavior. This is because the cell utilized the same active material. The resistance of the melt‐quenched amorphous state was lower than the typical range of ≈1 MΩ. This is because a small cell size and a thin PC layer was utilized. The amorphous region would be smaller and thus the resistance of the amorphous state is lower.

The crystallization process was slower than amorphization and it presented a switching time limitation of PCM. This time limitation of PCM had been previously investigated. In that study, the origin of the time limitation could be divided into two contributions: i) crystal‐nucleation limitation and ii) crystal‐growth limitation. The maximum crystallization rate of PCM was determined by the total rates for nucleation (Equation (1)) and growth (Equation (2))^[^ ^89^ ^]^

(1) $$I\left(t\right)=4f\left(1\right)\gamma n_{c}^{\frac{2}{3}}\mathrm{Zexp}\frac{16\pi v_{m}^{2}\sigma^{3}}{3\Delta g^{2}k_{B}T}{\left(\frac{4\pi \tau }{t}\right)}^{2}\exp\left(-\frac{\pi^{2}\tau }{4t}\right)$$

where *f*(1) is the concentration of GST molecules, *Z* is the Zeldovich factor, *γ* is the molecular jump frequency at the interface between amorphous and crystalline phase, *n*_c_ is the maximum number of GST molecules in the cluster, *v*_m_ is the volume of a GST “molecule”, *σ* is the interfacial energy, ∆*g* is the bulk free‐energy difference per GST molecule between amorphous and crystalline phases, *k*_B_ is the Boltzmann constant, *T* is the temperature, *τ* is the rate of molecular rearrangement during nucleation, and *t* is the time.

(2) $$\frac{\mathrm{dr}}{\mathrm{dt}}=\frac{16D}{\pi^{2}}{\left(\frac{3v_{m}}{4\pi }\right)}^{1/3}\sinh\left[\frac{1}{2k_{B}T}\left(\Delta g-\frac{2\sigma }{r}v_{m}\right)\right]$$

where *D* is the diffusion jump frequency of the GST molecules, *v*_m_ is the volume of a GST molecule, *k*_B_ is the Boltzmann constant, *T* is the temperature, ∆*g* is the bulk free‐energy‐difference per GST molecule between amorphous and crystalline phases, *σ* is the interfacial energy and *r* is the radius of a cluster.

## Conflict of Interest

The authors declare no conflict of interest.

## Supporting information

Supporting information

## Data Availability

The data that support the findings of this study are available from the corresponding author upon reasonable request.

## References

[1] N. Chakraborty , M. Mukherjee , J. Li , M. Shojafar , Y. Pan , IEEE Internet Things J. 2021, 9, 2614.

[2] X. Wang , Z. Yan , R. Zhang , P. Zhang , J. Netw. Comput. Appl. 2021, 188, 103080.

[3] Z. A. Collier , J. Sarkis , Int. J. Prod. Res. 2021, 59, 3430.

[4] A. Yeboah‐Ofori , S. Islam , E. Yeboah‐Boateng , in 2019 Int. Conf. Cyber Security and Internet of Things (ICSIoT), IEEE, Accra, Ghana 2019.

[5] P.‐Y. Lin , W.‐S. Lan , Y.‐H. Chen , W.‐C. Wu , Entropy 2022, 24, 284.35205577

[6] S. McGrew , Countermeasures against Hologram Counterfeiting, 2000. http://www.nli-ltd.com/publications/countermeasures.php

[7] J. M. Perkel , Nature 2017, 542, 125. 28150787 10.1038/542125a

[8] W. Shi , J. Cao , Q. Zhang , Y. Li , L. Xu , IEEE Internet Things J. 2016, 3, 637.

[9] K. Cao , Y. Liu , G. Meng , Q. Sun , IEEE Access 2020, 8, 85714.

[10] Y. Xiao , Y. Jia , C. Liu , X. Cheng , J. Yu , W. Lv , Proc. IEEE 2019, 107, 1608.

[11] M. Caprolu , R. Di Pietro , F. Lombardi , S. Raponi , in 2019 IEEE Int. Conf. on Edge Computing (EDGE), IEEE, Milan, Italy 2019.

[12] J. Zhang , B. Chen , Y. Zhao , X. Cheng , F. Hu , IEEE Access 2018, 6, 18209.

[13] S. Sidhu , B. J. Mohd , T. Hayajneh , J. Sens. Actuator Networks 2019, 8, 42.

[14] O. Demigha , R. Larguet , Comput. Secur. 2021, 103, 102117.

[15] Y. Gao , S. F. Al‐Sarawi , D. Abbott , Nat. Electron. 2020, 3, 81.

[16] A. P. James , Eur. Phys. J. Spec. Top. 2019, 228, 2301.

[17] C. E. Shannon , The Bell System Technic. J. 1949, 28, 656.

[18] S. Mrdovic , B. Perunicic , in Networks 2008‐The 13th Int. Telecommunications Network Strategy and Planning Symp, IEEE, Budapest, Hungary 2008.

[19] S. Mittal , A. I. Alsalibi , J. Hardware Syst. Secur. 2018, 2, 179.

[20] M. Mahmoodi , D. Strukov , O. Kavehei , IEEE Trans. Electron Devices 2019, 66, 5050.

[21] K. Shamsi , Y. Jin , in 2016 IEEE 34th VLSI Test Symp. (VTS), IEEE, Las Vegas, NV 2016.

[22] D. DiMase , Z. A. Collier , J. Carlson , R. B. Gray Jr , I. Linkov , Risk Anal. 2016, 36, 1834. 26800103 10.1111/risa.12536

[23] L. Aniello , B. Halak , P. Chai , R. Dhall , M. Mihalea , A. Wilczynski , Int. J. Inf. Secur. 2021, 20, 445.

[24] F. Tao , H. Zhang , A. Liu , A. Y. Nee , IEEE Trans. Industr. Inform. 2018, 15, 2405.

[25] M. Liu , S. Fang , H. Dong , C. Xu , J. Manuf. Syst. 2021, 58, 346.

[26] A. El Saddik , IEEE Multimedia 2018, 25, 87.

[27] M. Jacoby , T. Usländer , Appl. Sci. 2020, 10, 6519.

[28] Y. Gao , D. C. Ranasinghe , S. F. Al‐Sarawi , O. Kavehei , D. Abbott , IEEE Access 2016, 4, 61.

[29] R. Wang , J.‐Q. Yang , J.‐Y. Mao , Z.‐P. Wang , S. Wu , M. Zhou , T. Chen , Y. Zhou , S.‐T. Han , Adv. Intell. Syst. 2020, 2, 2000055.

[30] T. McGrath , I. E. Bagci , Z. M. Wang , U. Roedig , R. J. Young , Appl. Phys. Rev. 2019, 6, 011303.

[31] C. Herder , M.‐D. Yu , F. Koushanfar , S. Devadas , Proc. IEEE 2014, 102, 1126.

[32] S. Khalfaoui , J. Leneutre , A. Villard , I. Gazeau , J. Ma , P. Urien , Sensors 2021, 21, 8415. 34960505 10.3390/s21248415 PMC8705400

[33] Y. Pang , B. Gao , B. Lin , H. Qian , H. Wu , Adv. Electron. Mater. 2019, 5, 1800872.

[34] G. Rajendran , W. Banerjee , A. Chattopadhyay , M. M. S. Aly , Adv. Electron. Mater. 2021, 7, 2100536.

[35] P. Mall , R. Amin , A. K. Das , M. T. Leung , K.‐K. R. Choo , IEEE Internet Things J. 2022, 2022, 8998339.

[36] S.‐O. Park , H. Jeong , J. Park , J. Bae , S. Choi , Nat. Commun. 2022, 13, 2888. 35660724 10.1038/s41467-022-30539-6 PMC9166790

[37] A. Sebastian , M. L.e Gallo , R. Khaddam‐Aljameh , E. Eleftheriou , Nat. Nanotechnol. 2020, 15, 529. 32231270 10.1038/s41565-020-0655-z

[38] Y. Gao , D. C. Ranasinghe , S. F. Al‐Sarawi , O. Kavehei , D. Abbott , Sci. Rep. 2015, 5, 12785. 26239669 10.1038/srep12785 PMC4523939

[39] H. M. Ibrahim , H. Abunahla , B. Mohammad , H. AlKhzaimi , Sci. Rep. 2022, 12, 8633. 35606367 10.1038/s41598-022-11240-6 PMC9126908

[40] M. A. Zidan , J. P. Strachan , W. D. Lu , Nat. Electron. 2018, 1, 22.

[41] A. Mehonic , A. J. Kenyon , Nature 2022, 604, 255. 35418630 10.1038/s41586-021-04362-w

[42] L. Gao , Q. Ren , J. Sun , S.‐T. Han , Y. Zhou , J. Mater. Chem. C 2021, 9, 16859.

[43] Y. Li , Z. Wang , R. Midya , Q. Xia , J. J. Yang , J. Phys. D: Appl. Phys. 2018, 51, 503002.

[44] R. A. John , N. Shah , S. K. Vishwanath , S. E. Ng , B. Febriansyah , M. Jagadeeswararao , C.‐H. Chang , A. Basu , N. Mathews , Nat. Commun. 2021, 12, 3681. 34140514 10.1038/s41467-021-24057-0 PMC8211866

[45] H. Nili , G. C. Adam , B. Hoskins , M. Prezioso , J. Kim , M. R. Mahmoodi , F. M. Bayat , O. Kavehei , D. B. Strukov , Nat. Electron. 2018, 1, 197.

[46] R. Zhang , H. Jiang , Z. Wang , P. Lin , Y. Zhuo , D. Holcomb , D. Zhang , J. Yang , Q. Xia , Nanoscale 2018, 10, 2721. 29419836 10.1039/c7nr06561b

[47] W. Zhang , R. Mazzarello , M. Wuttig , E. Ma , Nat. Rev. Mater. 2019, 4, 150.

[48] D. Lencer , M. Salinga , M. Wuttig , Adv. Mater. 2011, 23, 2030. 21469218 10.1002/adma.201004255

[49] X. Li , H. Chen , C. Xie , D. Cai , S. Song , Y. Chen , Y. Lei , M. Zhu , Z. Song , Phys. Status Solidi Rapid Res. Lett. 2019, 13, 1800558.

[50] M. L.e Gallo , A. Sebastian , J. Phys. D: Appl. Phys. 2020, 53, 213002.

[51] I. Butun , A. Sari , P. Österberg , Sensors 2020, 20, 5729. 33050165 10.3390/s20205729 PMC7601476

[52] K. Myny , Nat. Electron. 2018, 1, 30.

[53] L. Li , A. M. Belcher , D. K. Loke , Nanoscale 2020, 12, 24214. 33289758 10.1039/d0nr07320b

[54] B.‐S. Lee , R. M. Shelby , S. Raoux , C. T. Retter , G. W. Burr , S. N. Bogle , K. Darmawikarta , S. G. Bishop , J. R. Abelson , J. Appl. Phys. 2014, 115, 063506.

[55] V. E. Madhavan , M. Carignano , A. Kachmar , K. S. Sangunni , Sci. Rep. 2019, 9, 12985. 31506508 10.1038/s41598-019-49168-z PMC6737191

[56] B.‐S. Lee , K. Darmawikarta , S. Raoux , Y.‐H. Shih , Y. Zhu , S. G. Bishop , J. R. Abelson , Appl. Phys. Lett. 2014, 104, 071907.

[57] S. Choi , B. J. Choi , T. Eom , J. H. Jang , W. Lee , C. S. Hwang , J. Phys. Chem. C 2010, 114, 17899.

[58] W. Ren , B. Liu , B. Bao , Z. Song , Surf. Coat. Technol. 2018, 353, 309.

[59] C. Liu , H. Chen , S. Wang , Q. Liu , Y.‐G. Jiang , D. W. Zhang , M. Liu , P. Zhou , Nat. Nanotechnol. 2020, 15, 545. 32647168 10.1038/s41565-020-0724-3

[60] Y. K. Lee , C. Yoo , W. Kim , J. W. Jeon , C. S. Hwang , J. Mater. Chem. C 2021, 9, 3708.

[61] C. Qiao , Y. Guo , F. Dong , J. Wang , H. Shen , S. Y. Wang , M. Xu , X. Miao , Y. Zheng , R. Zhang , J. Mater. Chem. C 2018, 6, 5001.

[62] T. H. Lee , D. Loke , K. J. Huang , W. J. Wang , S. R. Elliott , Adv. Mater. 2014, 26, 7493. 25302744 10.1002/adma.201402696 PMC4557048

[63] J. Orava , A. L. Greer , Acta Mater. 2017, 139, 226.

[64] S. Lombardo , E. Rimini , M. Grimaldi , S. Privitera , Microelectron. Eng. 2010, 87, 294.

[65] S. Privitera , S. Lombardo , C. Bongiorno , E. Rimini , A. Pirovano , J. Appl. Phys. 2007, 102, 013516.

[66] M. A. Luong , N. Cherkashin , B. Pécassou , C. Sabbione , F. Mazen , A. Claverie , Nanomaterials 2021, 11, 1729. 34209198 10.3390/nano11071729 PMC8308197

[67] Y. Lai , B. Qiao , J. Feng , Y. Ling , L. Lai , Y. Lin , T. a. Tang , B. Cai , B. Chen , J. Electron. Mater. 2005, 34, 176.

[68] C. Li , C. Hu , J. Wang , X. Yu , Z. Yang , J. Liu , Y. Li , C. Bi , X. Zhou , W. Zheng , J. Mater. Chem. C 2018, 6, 3387.

[69] M. A. Luong , D. Wen , E. Rahier , N. Ratel Ramond , B. Pécassou , Y. Le Friec , D. Benoit , A. Claverie , Phys. Status Solidi Rapid Res. Lett. 2021, 15, 2000443.

[70] H. Ning , F. Farha , A. Ullah , L. Mao , IET Circuits Devices Syst. 2020, 14, 407.

[71] A. Chen , Solid‐State Electron. 2016, 125, 25.

[72] N. Shah , M. Alam , D. P. Sahoo , D. Mukhopadhyay , A. Basu , in Proc. of the 24th Asia and South Pacific Design Automation Conf, Association for Computing Machinery, Tokyo, Japan 2019.

[73] N. Shah , D. Chatterjee , B. Sapui , D. Mukhopadhyay , A. Basu , IEEE J. Emerging Sel. Top. Circuits Syst. 2021, 11, 319.

[74] A. Vijayakumar , V. C. Patil , C. B. Prado , S. Kundu , in 2016 IEEE Int. Symp. on Hardware Oriented Security and Trust (HOST), IEEE, McLean, VA 2016.

[75] L. Zhang , Z. H. Kong , C.‐H. Chang , A. Cabrini , G. Torelli , IEEE Trans. Inf. Forensics Secur. 2014, 9, 921.

[76] Y. Pang , B. Gao , D. Wu , S. Yi , Q. Liu , W.‐H. Chen , T.‐W. Chang , W.‐E. Lin , X. Sun , S. Yu , in 2019 IEEE Int. Solid‐State Circuits Conf. (ISSCC), IEEE, San Francisco, CA 2019.

[77] M. Rizzi , N. Ciocchini , A. Montefiori , M. Ferro , P. Fantini , A. L. Lacaita , D. Ielmini , IEEE Trans. Electron Devices 2015, 62, 2205.

[78] Z. Woods , J. Scoggin , A. Cywar , A. Gokirmak , IEEE Trans. Electron Devices 2017, 64, 4472.

[79] A. Aladool , M. Aziz , D. Wright , Phys. Status Solidi 2019, 256, 1800583.

[80] P. Guo , A. M. Sarangan , I. Agha , Appl. Sci. 2019, 9, 530.

[81] S. Raoux , H.‐Y. Cheng , M. Caldwell , H.‐S. Wong , Appl. Phys. Lett. 2009, 95, 071910.

[82] X. Wei , L. Shi , R. Walia , T. Chong , R. Zhao , X. Miao , B. Quek , IEEE Trans. Electron Devices 2005, 53, 56.

[83] C. Liu , X. Yan , X. Song , S. Ding , D. W. Zhang , P. Zhou , Nat. Nanotechnol. 2018, 13, 404. 29632398 10.1038/s41565-018-0102-6

[84] D. Loke , J. M. Skelton , W.‐J. Wang , T.‐H. Lee , R. Zhao , T.‐C. Chong , S. R. Elliott , Proc. Natl. Acad. Sci. U. S. A. 2014, 111, 13272. 25197044 10.1073/pnas.1407633111 PMC4169905

[85] D. Loke , T. Lee , W. Wang , L. Shi , R. Zhao , Y. Yeo , T. Chong , S. Elliott , Science 2012, 336, 1566. 22723419 10.1126/science.1221561

[86] D. K. Loke , J. M. Skelton , T. H. Lee , R. Zhao , T.‐C. Chong , S. R. Elliott , ACS Appl. Mater. Interfaces 2018, 10, 41855. 30507141 10.1021/acsami.8b16033

[87] M. Breitwisch , T. Nirschl , C. Chen , Y. Zhu , M. Lee , M. Lamorey , G. Burr , E. Joseph , A. Schrott , J. Philipp , in 2007 IEEE Symp. on VLSI Technology, IEEE, Kyoto, Japan 2007.

[88] H. Horii , J. Yi , J. Park , Y. Ha , I. Baek , S. Park , Y. Hwang , S. Lee , Y. Kim , K. Lee , in 2003 Symposium on VLSI Technology, Digest of Technical Papers (IEEE Cat. No. 03CH37407) IEEE, 2003, 177–178.

[89] K. F. Kelton , A. L. Greer , Nucleation in Condensed Matter: Applications in Materials and Biology, Elsevier, Amsterdam, Netherlands 2010.

