# Supplementary material for: Ultrafast Near‐Ideal Phase‐Change Memristive Physical Unclonable Functions Driven by Amorphous State Variations
Source: Adv Sci (Weinh). 2022 Nov 13;9(36):2204453. doi: 10.1002/advs.202204453 (PMC9798968; doi:10.1002/advs.202204453)
Supplement: Supplementary file 1 — Supporting information [file ADVS-9-2204453-s001.pdf]

Supporting Information

Ultrafast near-ideal phase-change memristive physical unclonable functions driven by amorphous state variations

Shao-Xiang Go,[1] Qiang Wang,[1] K. G. Lim,[1] Tae Hoon Lee,[2,3] Natasa Bajalovic,[1] Desmond K. Loke[1]

[1]*Department of Science, Mathematics and Technology, Singapore University of Technology and Design, Singapore 487372, Singapore*

[2]*Department of Engineering, University of Cambridge, Trumpington Street, Cambridge CB2 1PZ, UK*

[3]*School of Materials Science and Engineering, Kyungpook National University, Daegu 41566, Republic of Korea*

[*]*Correspondence and requests for material should be addressed to D.K.L (e-mail: desmond_loke@sutd.edu.sg).*

Table S1. National Institute of Science and Technology (NIST) tests. Results of NIST test for strong PUF construction. 20 million bits corresponding to 20 million CRPS were collected for the NIST test. The responses successfully pass all 15 tests.

Test	P-value	No. of runs	Pass No.	Pass
Frequency	0.358641	200	195	Pass
Block frequency	0.484646	200	197	Pass
Cumulative sums	0.167184	200	196	Pass
Runs	0.668321	200	198	Pass
Longest run	0.779188	200	198	Pass
FFT	0.213309	200	197	Pass
Serial	0.122325	200	200	Pass
Linear complexity	0.978072	200	197	Pass
Rank	0.410055	200	197	Pass
Approximate entropy	0.118812	200	197	Pass
Random excursions	0.141256	25	25	Pass
Random excursions variant	0.021262	25	25	Pass
Non-overlapping template	0.002758	200	198	Pass
Overlapping template	0.616000	200	198	Pass
Universal test	0.739918	20	20	Pass

Table S2. Reference utilized for comparison of mean value of HD distribution for UQ for weak PUF (Figure S10).

Ref no.	Reference
1	Liu, R., Wu, H., Pang, Y., Qian, H., & Yu, S. (2016, May). A highly reliable and tamper-resistant RRAM PUF: Design and experimental validation. In *2016 IEEE International Symposium on Hardware Oriented Security and Trust (HOST)* (pp. 13-18). IEEE.
2	John, R. A., Shah, N., Vishwanath, S. K., Ng, S. E., Febriansyah, B., Jagadeeswararao, M., Chang, C.-H., Basu, A., & Mathews, N.. (2021). Halide perovskite memristors as flexible and reconfigurable physical unclonable functions.
3	Li, J., Cui, Y., Gu, C., Wang, C., Liu, W., & Lombardi, F. (2021). A physical unclonable function using a configurable tristate hybrid scheme with non-volatile memory. *IEEE Open Journal of Nanotechnology*, *2*, 31-40.

Table S3. Reference utilized for comparison of mean value of HD distribution for UF for weak PUF (Figure S11).

Ref no.	Reference
1	John, R. A., Shah, N., Vishwanath, S. K., Ng, S. E., Febriansyah, B., Jagadeeswararao, M., Chang, C.-H., Basu, A., & Mathews, N.. (2021). Halide perovskite memristors as flexible and reconfigurable physical unclonable functions.
2	Lin, B., Pang, Y., Gao, B., Tang, J., Wu, D., Chang, T. W., … & Wu, H. (2021). A highly reliable RRAM physically unclonable function utilizing post-process randomness source. *IEEE Journal of Solid-State Circuits*, *56*(5), 1641-1650.
3	Li, J., Cui, Y., Gu, C., Wang, C., Liu, W., & Lombardi, F. (2021). A physical unclonable function using a configurable tristate hybrid scheme with non-volatile memory. *IEEE Open Journal of Nanotechnology*, *2*, 31-40.

Table S4. Reference utilized for comparison of number of NIST tests passed (Figure S12).

Ref no.	Reference
1	Ali, R., Wang, Y., Ma, H., Hou, Z., Zhang, D., Deng, E., & Zhao, W.. (2021). *A Reconfigurable Arbiter PUF Based on STT-MRAM*.
2	Pang, Y., Gao, B., Wu, D., Yi, S., Liu, Q., Chen, W. H., ... & Wu, H. (2019, February). 25.2 A reconfigurable RRAM physically unclonable function utilizing post-process randomness source with< 6× 10− 6 native bit error rate. In *2019 IEEE International Solid-State Circuits Conference-(ISSCC)* (pp. 402-404). IEEE
3	Govindaraj, R., Ghosh, S., & Katkoori, S.. (2020). Design, Analysis and Application of Embedded Resistive RAM Based Strong Arbiter PUF. *IEEE Transactions on Dependable and Secure Computing*, *17*(6), 1232–1242.

Table S5. Reference utilized for comparison of maximum attack accuracy on strong PUF (Figure S13).

Ref no.	Reference
1	Wang, Y., Huo, Q., Xu, X., Tan, F., Gao, R., Luo, Q., Yang, Y., Ren, Q., Zhao, X., Wang, X., Lei, D., & Zhang, F.. (2021). A Homogeneous, Reconfigurable, and Efficient Implementation of PUF in 3-D Selector-Free RRAM. *IEEE Transactions on Electron Devices*, *68*(5), 2577–2581
2	John, R. A., Shah, N., Vishwanath, S. K., Ng, S. E., Febriansyah, B., Jagadeeswararao, M., Chang, C.-H., Basu, A., & Mathews, N.. (2021). Halide perovskite memristors as flexible and reconfigurable physical unclonable functions.
3	Pang, Y., Gao, B., Wu, D., Yi, S., Liu, Q., Chen, W. H., ... & Wu, H. (2019, February). 25.2 A reconfigurable RRAM physically unclonable function utilizing post-process randomness source with< 6× 10− 6 native bit error rate. In *2019 IEEE International Solid-State Circuits Conference-(ISSCC)* (pp. 402-404). IEEE

Table S6. Reference utilized for comparison of mean value of HD distribution for reconfiguration (Figure S14).

Ref no.	Reference
1	John, R. A., Shah, N., Vishwanath, S. K., Ng, S. E., Febriansyah, B., Jagadeeswararao, M., Chang, C.-H., Basu, A., & Mathews, N.. (2021). Halide perovskite memristors as flexible and reconfigurable physical unclonable functions.
2	Pang, Yachuan, et al. "25.2 A reconfigurable RRAM physically unclonable function utilizing post-process randomness source with< 6× 10− 6 native bit error rate." *2019 IEEE International Solid-State Circuits Conference-(ISSCC)*. IEEE, 2019.
3	Lu, L., Chen, Y. Z., & Kim, T. T. H. (2021, May). A Configurable Randomness Enhanced RRAM PUF with Biased Current Sensing Scheme. In *2021 IEEE International Symposium on Circuits and Systems (ISCAS)* (pp. 1-5). IEEE.

Table S7. Reference utilized for comparison of stimulus length for reconfiguration (Figure S15).

Ref no.	Reference
1	Lin, B., Gao, B., Pang, Y., Chen, B., Tang, J., Qian, H., & Wu, H. (2020, April). A novel bi-functional memory-PUF module utilizing adjustable switching window of RRAM. In *2020 4th IEEE Electron Devices Technology & Manufacturing Conference (EDTM)* (pp. 1-4). IEEE.
2	Pang, Y., Gao, B., Wu, D., Yi, S., Liu, Q., Chen, W. H., ... & Wu, H. (2019, February). 25.2 A reconfigurable RRAM physically unclonable function utilizing post-process randomness source with< 6× 10− 6 native bit error rate. In *2019 IEEE International Solid-State Circuits Conference-(ISSCC)* (pp. 402-404). IEEE.
3	John, R. A., Shah, N., Vishwanath, S. K., Ng, S. E., Febriansyah, B., Jagadeeswararao, M., Chang, C.-H., Basu, A., & Mathews, N.. (2021). Halide perovskite memristors as flexible and reconfigurable physical unclonable functions.

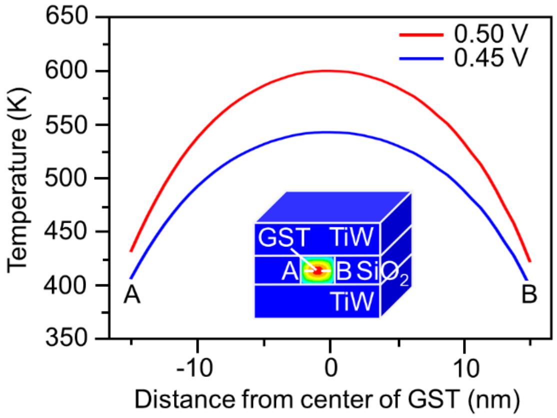

Figure S1. Temperature profiles along the line A–B in the A PUF cell for different stimulus voltages.

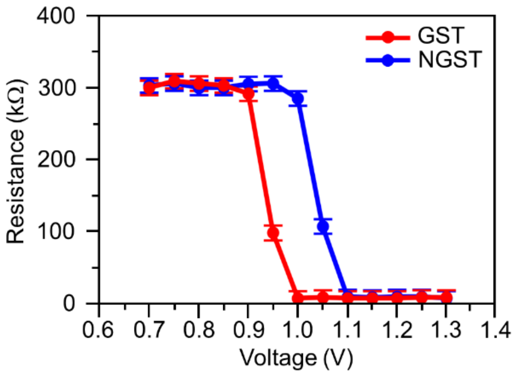

Figure S2. Variation of resistance of A PUF cell for different stimulus voltages and for different material types. The error bars represent the range of values from experiments performed on three different cells in a batch.

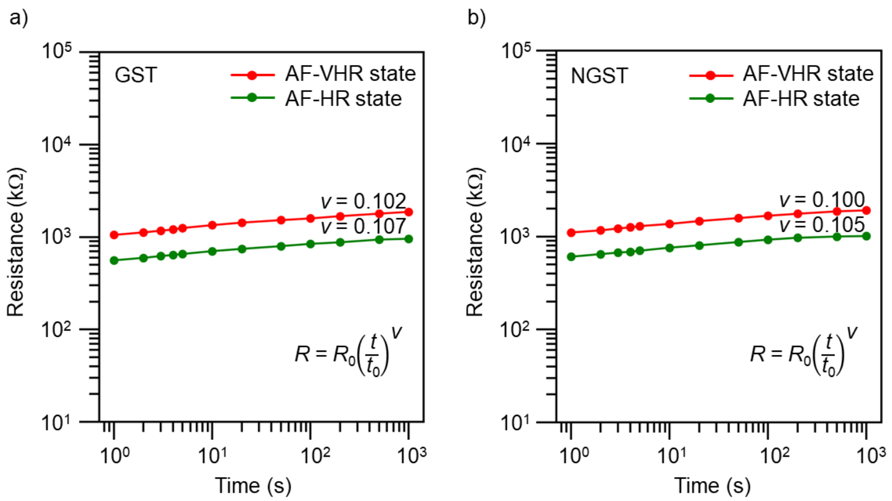

Figure S3. Time evolution of resistance of A PUF cell for different material states and for different material types a) GST and b) NGST.

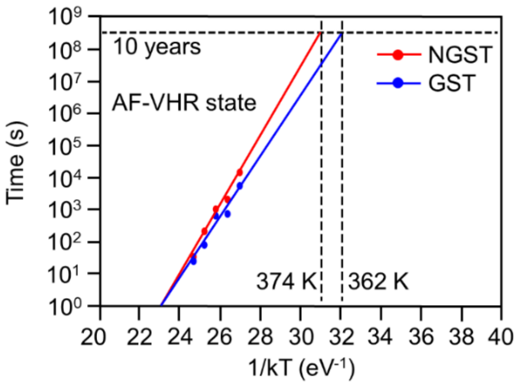

Figure S4. An Arrhenius extrapolation for the 10-year data retention of the A PUF cell in the very high resistance as-fabricated state for different material types.

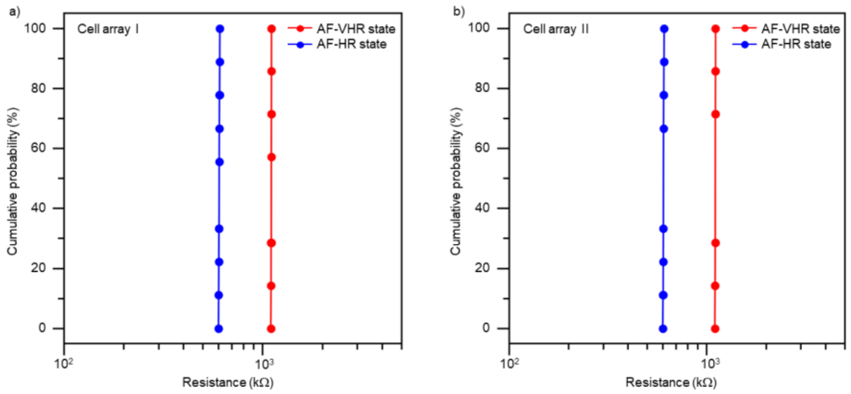

Figure S5. Cumulative probability distribution plots of the A PUF cells in the as-fabricated very high resistance state (red) and as-fabricated high resistance state (blue) and for the a) cell array I and b) cell array II.

Weak PUF To implement the weak PUF, the cells in the very high/ high resistance as-fabricated state was characterized. The variation that occurs in the fabrication process is utilized as the variation source, where cells either in the very high resistance as-fabricated state (~1100 kΩ/ 0.90 μS) or high resistance as-fabricated state (~600 kΩ/ 1.60 μS) are induced with an almost equal probability. Since the resistance of the cells in the very high resistance as-fabricated state is almost 2 times larger than that of the cells in the high resistance as-fabricated state, this justifies the choice of fabrication process as the variation source due to larger sensing window.

For PUF performed by traditional nonvolatile devices, if the challenge condition results in a device with the low conductance state, the response is ‘0’, and the response becomes ‘1’ for the case where the challenge condition leads to a device with the high conductance state [1, 2]. Moreover, digital keys/ bits are generated using an array of cells for conventional nonvolatile devices [3]. Based on this protocol, we created the A PUF. This is achieved by utilizing an appropriate choice of the reference conductance $G_{ref,1}$ (1.25 μS), which is the average of the cell conductance for the very high resistance as-fabricated state, and the cell conductance for high

resistance as-fabricated state. In the weak PUF protocol, each n-th response bit $b_r^{(n)}$ is generated from the n-th challenge $C^{(n)}$, which defines the 8 different positions 8 cells should be chosen from the group of 16 different cells. The response bit is '1' if at least 4 out of 8 selected cells are cells in the very high resistance as-fabricated state (~1100 kΩ/ 0.9 μS) and '0' otherwise. This is attained by utilizing the bit counter to count the number of cells with a conductance below the $G_{\text{ref},1}$ out of 8 cells. If the count is greater than or equal to 4, cells in the very high resistance as-fabricated state occurs for at least 4 out of 8 selected cells.

In this weak PUF methodology, nC_8 distinct challenges are defined, where n is the total number of cells utilized. We selected 64 different challenges out of a total of 12870 challenges to generate the 64-bit key from corresponding response bits. Moreover, the group of 64 challenges is fixed to create the key, and response bits are generated using reading operation only. By utilizing an appropriate reading voltage and carefully chosen $G_{\text{ref},1}$ with a sufficient margin between the $G_{\text{ref},1}$ and the cell conductance for the very high/ high resistance as-fabricated state (the margin is ±0.35 μS), we can avoid unintended programming of the cell, and this ensures maximal reliability. Moreover, since this methodology does not utilize any write operations, it alleviates the problem of limited writing endurance that may be observed in traditional PUF based on memristive devices.

To validate the security strength of the weak A-PUF, we investigated important metrics such as uniqueness (UQ) and uniformity (UF). The UQ is defined by the hamming distance (HD) between the responses of different PUF cells when subjected to the same challenge with an ideal inter-HD of 50%, while the UF is described by the percentage of bit '1' or '0' in the response bit string with an optimal value of 50%. These metrics are estimated by counting the number of cells in the very high/ high resistance as-fabricated state and computing inter-HD. We omitted the

calculation of intra-HD since the contrast between the cell conductance for very-high-resistance as-fabricated state and cell conductance for high-resistance as-fabricated state is sufficiently large. By utilizing an appropriate reading voltage and $G_{ref,1}$, reading errors are avoided, thus ensuring high reliability (intra-HD of 0%). To evaluate the UQ, we rearranged the 4 × 4 cell array by randomly shuffling the position of 16 cells for every new group. For each group, we generated a 64-bit key using the same circuit and the fixed set of 64 challenges, followed by calculating the HD of $\binom{8}{2}$ = 28 pairs of keys. Here, we obtained a mean value of 50.89% and standard deviation of 9.06% (Figure 3e). To estimate the UF, 8 groups of 64 bits are sequentially generated and the UF is calculated for each group. The average UF of 50.00% is attained with a standard deviation of 16.24% (Figure 3f). These results show that if the attacker has access to the resistance distribution and challenge set utilized in this work, by maintaining secrecy of the orientation of the 16 cells, it is unlikely that the attacker will be able to generate the same key since many possible arrangements (2^{16}) exist. Furthermore, the same challenge might result in different selections of 8 cells from the 16-cell configuration since cells in different positions are harnessed.

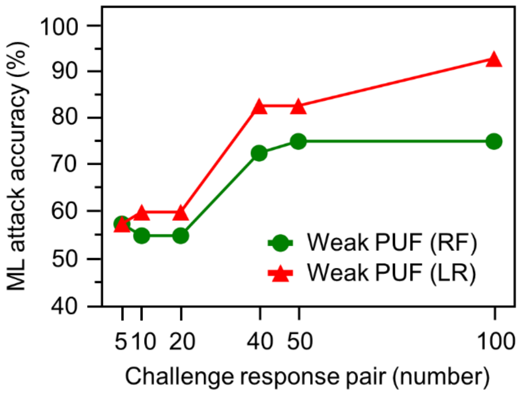

Figure S6. Weak-A-PUF ML-attack-accuracy variation for different number of CRPs used for training and for different ML algorithms, random forest (RF) and logistic regression (LR).

Security of weak PUF We investigated the ML attack resilience of weak PUF. A total of 16 C_8 = 12870 CRPs were generated in the implementation of the weak PUF with 16 cells. ML attacks are tested on the weak A PUF using two different models, logistic regression (LR) and random forest (RF). In this work, we generated 140 CRPs from weak PUF. 100 CRPs are utilized for training, and for testing, a fixed test set of 40 CRPs is harnessed. The number of training samples is varied between 5 to 100 with the same 40 CRPs used as test set. We obtained a maximum attack accuracy of ~75% and 92.5% using the RF and LR models and with the utilization of 100 CRPs for training, respectively, illustrating the vulnerability of this implementation of weak PUF. For instance, if the relationship between any of the 9 sets of 8 cells chosen from 9 distinct cells is disclosed (9 C_8 = 9), the attacker is able to deduce resistances of all 9 cells in this configuration by solving a system of 9 linear equations with 9 unknowns. Similarly, the relationship between different groups of cells created from 16 cells becomes easily known using a small number of CRPs. This is a possible limitation which can be avoided if the CRP relationship is kept secret or the keys are reconfigured when necessary to alter CRP space.

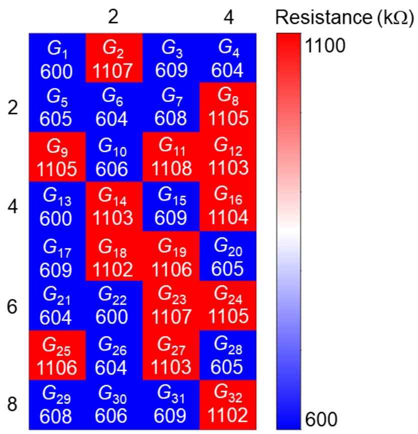

Figure S7. Heatmap of the resistance values of cells with VHR/ HR as-fabricated states demonstrating a high degree of randomness.

Strong PUF We then implement a strong PUF by reading the resistance of A PUF cells efficiently via in-memory computing to obtain a large number of CRPs. To achieve an increased number of CRPs, an extended challenge-bit string is utilized. This is made possible by utilizing an increasing number of cells in the very high/ high resistance as-fabricated state (8 × 4 array = 32 cells) and a specialized circuit.

Figure 4a–c illustrates the flowchart for the generation of keys from the strong PUF array of 32 cells in the very high/ high resistance as-fabricated state, and the circuit harnessed in this implementation. For each challenge $C^{(n)}$, the corresponding response bit $b_r^{(n)}$ is generated via a specified operation f based on 2 conditions: (i) pairing up cells with their immediate neighbors and comparing the difference in conductance for each cell pair to an appropriate reference conductance $G_{\text{ref},2}$ (0.35 μS), and (ii) reading conductance of odd-indexed cells and comparing the odd-indexed cell conductance to the $G_{\text{ref},1}$ (1.25 μS) (the same reference conductance is utilized in weak PUF). The overall procedure for the generation of a response bit from a specified challenge can be explained mathematically as:

$$b_{r_i}^{(n)} = 1 \quad \text{if } C_i^{(n)} = 1 \text{ \& } \left| G_{2i} - G_{2i-1} \right| > 0.35\ \mu\text{S}, \quad i = 1, 2, 3, \dots, 16 \tag{1}$$
$$= 0 \quad \text{otherwise}$$

$$b_{r_{2j+1}}^{(n)} = 1 \quad \text{if } C_{j+17}^{(n)} = 1 \text{ \& } G_{2j+1} < 1.25\ \mu\text{S}, \quad j = 0, 1, 2, \dots, 15 \tag{2}$$
$$= 0 \quad \text{otherwise}$$

$$b_r^{(n)} = 1 \quad \text{if } \left(\sum_{i=1}^{16} b_{r_i}^{(n)} + \sum_{j=0}^{15} b_{r_{2j+1}}^{(n)} \right) \text{ is odd}, \tag{3}$$
$$= 0 \quad \text{otherwise}$$

where $C_i^{(n)}$ represents the *i*-th bit in the *n*-th challenge and $b_{r_i}^{(n)}$ describes the corresponding intermediate response bit. If the $C_i^{(n)} = 1$, viz., the *i*-th switch is turned on, the intermediate response bit '1' results depending on defined conditions. In Equation (1), if the $C_i^{(n)} = 1$ (a turned-on *i*-th switch) and for the corresponding cell pair with the conductance G_{2i}, G_{2i-1}, in the case where one cell in the very high resistance as-fabricated state and the other cell in the high resistance as-fabricated state occurs, an intermediate response bit '1' is induced. For Equation (2), the intermediate response bit '1' manifests, if the $C_i^{(n)} = 1$, i.e., the *i*-th switch is turned on, and the corresponding odd-indexed cell in the very high resistance as-fabricated state is disclosed. Finally, in Equation (3), the intermediate response bits resulting from all bits of the challenge $C^{(n)}$ are summed up and the parity of this sum is evaluated. The final response bit $b_r^{(n)}$ from the *n*-th challenge $C^{(n)}$ is '1' if the sum is odd, otherwise '0'. This is implemented using XOR cascade (Figure 4a). Note that if the $C_i^{(n)} = 0$, conditions for the cells are not examined. Thus, the *i*-th intermediate response bit is not generated. By pairing up cells with immediate neighbors and evaluating the conductance of odd-indexed cells, this enables high independence among intermediate response bits and avoid information leakage. For example, in

the pair of cells with the conductance G_{2i}, G_{2i-1}, for the case of an attacker that has access to information about the conductance of the odd-indexed cell G_{2i-1}, the attacker does not know if the conductance of the neighboring cell G_{2i} exhibits the same value. Thus, Equations (1), (2) for this cell pair results in independent intermediate-response-bits. In this implementation, the number of bits in the challenge is 32 (corresponding to 32 switches in the circuit), resulting in $2^{32} = 4 \times 10^9$ CRPs. Figure 4c shows the schematic illustration of the generation of a key with 10 × 10 bits from strong PUF setup. This is achieved by administering a group of 100 different 32-bit challenge to the strong-PUF system and consolidating the 100 generated response bits in the form of a response matrix, which is utilized as the key for authentication purposes. Since the CRP space is large, it is possible to generate many different keys with wide bit-array-range.

We investigated the resilience of the strong PUF from machine learning (ML) attacks. In a ML attack, the machine learner is assumed to have access to CRPs and is able to utilize different algorithms to model CRP relationships. ML attacks are tested on the A PUF with two different algorithms, logistic regression (LR) and multi-layered perceptron (MLP). In this work, a total of 1.4×10^6 CRPs from the strong PUF are generated. We utilized an upper bound of 10^6 CRPs for training and a fixed test set of 0.4×10^6 CRPs for testing. The number of training samples is varied between 10^2 and 10^6, and the same CRP test-set is harnessed. We obtained an attack accuracy of ~50%, close to the 50% accuracy of random guessing. Moreover, the attack accuracy remains the same with an increased number of CRPs utilized for training (Figure 4d). In the worst-case scenario, if the attacker attains a CRP number above 10^6 CRPs, it still causes a strain on attacker computation resources. This could be considered as a universal approach to design ML-resistant strong PUF using small to medium-sized arrays through memristive materials and with few tens of cells.

To assess the security strength of this strong PUF, we performed simulations to generate 8 different instances of the $8 \times 4 = 32$ cells, corresponding to 8 random different resistance distributions of 32 cells in very high/ high resistance as-fabricated states. The idea is that in the worst-case scenario, if the information about the utilization of 32 cells becomes accessible to the attacker, the attacker does not know the location of the cells in very high resistance as-fabricated state and cells in high resistance as-fabricated state. Even if the attacker tries to fabricate 32 cells to replicate the strong PUF, it is unlikely that the attacker will be able to obtain the same distribution of cells in the very high/ high resistance as-fabricated state, and attain different keys when the attacker utilizes the same group of challenges (similar to the argument used in weak PUF). We analyzed the UQ by consolidating the 100 response bits (this forms a 100-bit key) generated using 100 challenges (1 challenge results in 1 response bit) for 8 different instances, and then calculating the inter-HD for $\binom{8}{2} = 28$ pairs of 100-bit keys. A mean inter-HD value of 50.66% with a standard deviation of 5.83% is achieved (Figure 4e), illustrating that the strong PUF harnessed in this work is unclonable and unique. Moreover, we sequentially organized the response bits into 64 groups (each group contains 8 response bits) and examined the UF for strong PUF. The mean value for UF is 50.00% with a standard deviation of 16.38% (Figure 4f), indicating that the generated bitstream has minimal bias in strong PUF. To evaluate the randomness of bitstreams generated from the strong PUF, we consolidated groups of at least 10^5 response bits and passed them through all 15 tests of the National Institute of Science and Technology (NIST) test suite (Table S1). The response bits passed all 15 tests, exhibiting a high quality of randomness of CRPs.

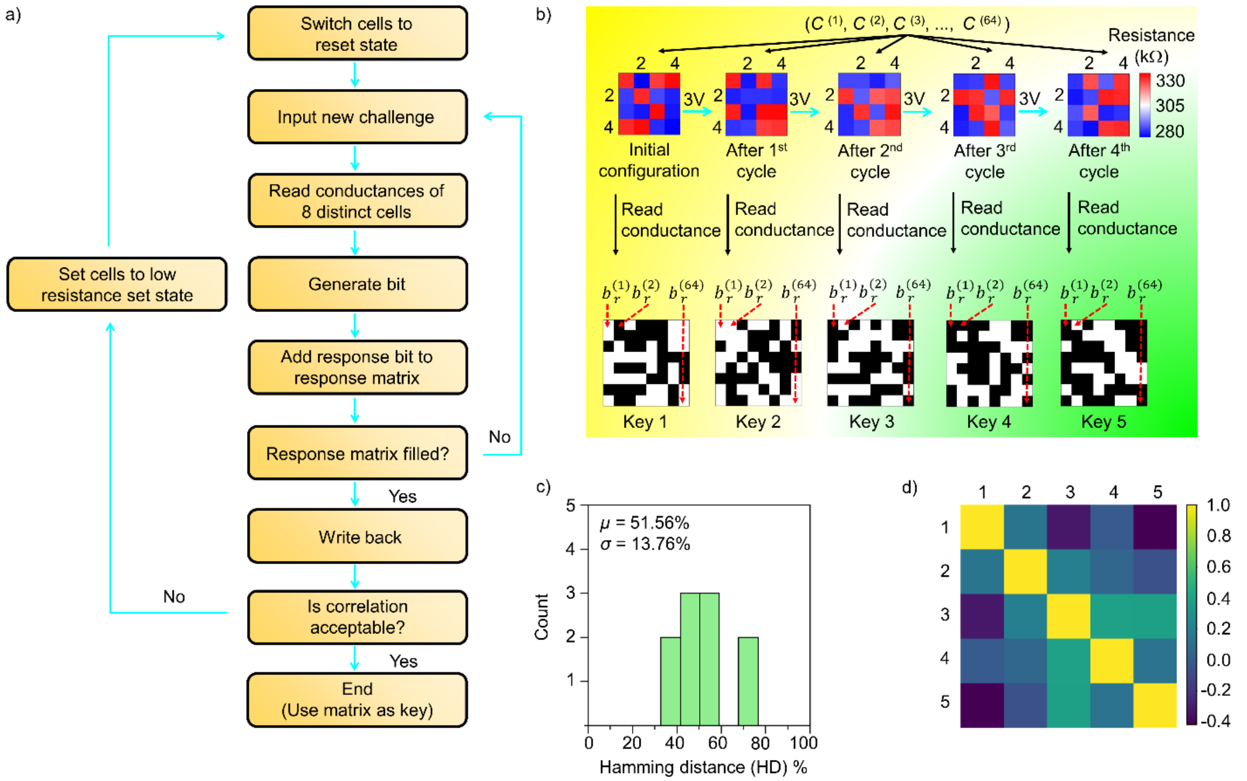

Figure S8. Reconfigurable A PUF. a) Reconfiguration flowchart showing how reconfiguration is performed using the cycle-to-cycle variability by resetting the cell to the reset state and using the same protocol again every cycle to generate fresh keys. If the correlation factor between the new key and the key from previous cycle is not sufficiently small, the PUF is repeatedly reconfigured. The reconfiguration protocol is similar to the weak PUF protocol. **b)** Analog color map of resistances of 16 cells and the digital keys after every new cycle. The $8 \times 8 = 64$-bit keys corresponding to different cycles are generated from the same group of 64 challenges and the same protocol. **c)** Histogram of the reconfiguration HD obtained by calculating the HD for $\binom{5}{2} = 10$ pairs of $8 \times 8 = 64$ bit keys, with a mean value of 51.56% illustrating good independence of key bits for different cycles. **d)** Correlation matrix for 5 cycles of reconfiguration with an average correlation of 0.17.

Reconfiguration The reconfiguration of the PUF key is required for supporting ownership change, changes in software versions or preventing external attacks by malicious third parties [4, 5]. We harnessed the cycle-to-cycle variability of PCM to switch the cells into new reset states to facilitate the freshness of cryptographic key. With this reconfiguration, the trustworthy party can now reconfigure the keys, rendering all previous keys inferred or known by the attackers futile. This is achieved by applying 3.5 V pulses to switch all the 16 cells from the low resistance crystallized state (~10 kΩ/ 100 μS) to the high resistance melt-quenched A state (~300 kΩ/ 3.3 μS). Administering these pulses to the same cells for 5 different cycles results in a stochastic variation in the resistance slightly above/ below the mean resistance of 300 kΩ (equivalently, the conductance slightly above/ below the mean conductance of 3.33 μS). This forms the basis for reconfiguration. The reference conductance $G_{ref,3}$ (3.33 μS) is utilized. The difference between the reference conductance of the reconfigured weak PUF and that of the original weak PUF is that the conductance value is changed from 1.25 μS to 3.33 μS. Supporting Information Figure S8c shows the histogram of reconfiguration HD. This is obtained by calculating the HD for $\binom{5}{2} = 10$ pairs of $8 \times 8 = 64$-bit keys for 5 different configurations/ cycles. A mean value of 51.56% with a standard deviation of 13.76% is achieved. Moreover, the cross-correlation between reconfigured keys discloses small values and with a mean value of 0.17 (Figure S8d). These indicate good independence between reconfigured keys.

Write back of weak/ strong/ reconfigured PUF In weak PUF, the spontaneous crystallization effect might affect the counting of cells with a conductance above the G_{ref} if cells in the as-fabricated very-high-resistance state are utilized for a specified number of cells out of 8 arbitrary

chosen cells. This might result in unwanted bit flips after the response matrix (to be used as the key) is filled. To ensure that the same bit and response matrix (key) is generated subsequently after accounting for spontaneous crystallization effects, we adopt the write back strategy in this setting. The write back procedure comprises setting all the cells in the as-fabricated very-high-resistance state (~1100 kΩ/ 0.9 μS) to the new low resistance value of ~10 kΩ/ 100 μS, while the cells in the as-fabricated high resistance state (~600 kΩ/ 1.6 μS) remain unchanged. After the write back procedure, the cells in very high resistance/ high resistance states (~1100 kΩ, 0.9 μS/ ~600 kΩ, 1.6 μS) become the cells in new low resistance/ high resistance states (~10 kΩ, 100 μS/ ~600 kΩ, 1.6 μS). This means that the circuit logic needs to be modified as well. To select the new reference conductance $G_{\mathrm{ref},4}$, we calculated the average of conductance in the new low resistance state/ high resistance state (100 μS/ 1.6 μS). Thus, the new $G_{\mathrm{ref},4}$ of 50 μS is chosen. If the count from the bit counter is greater than or equal to 4, i.e., cells in the new low resistance state results for at least 4 out of 8 selected cells, the output bit '1' is generated, '0' otherwise. This preserves the reliability of weak PUF.

In strong PUF, the spontaneous crystallization effect may influence the pairwise comparison output bit in Equation (1). For instance, if two cells in the as-fabricated very high resistance state are utilized (~1100 kΩ/ 0.9 μS), the pairwise comparison in Equation (1) results in intermediate output bit '0'. However, due to spontaneous crystallization, the cells may switch from the as-fabricated very high resistance state to the low resistance state, and exhibit an absolute value of conductance difference above the original $G_{\mathrm{ref},2}$ of 0.35 μS. The intermediate output bit '1' results and a bit flip occurs. To avoid this, we propose to utilize the write back procedure by setting the cells from the as-fabricated very high resistance state (~1100 kΩ/ 0.9 μS) to the new low resistance value of ~10 kΩ/ 100 μS, while the cells in the as-fabricated high

resistance state (~600 kΩ/ 1.6 μS) remain the same. This requires modifying the circuit logic of strong PUF after the response matrix is generated. The Equation (1) is kept fixed since the new difference in the conductance is at least 98.4 μS (i.e., 100 μS − 1.6 μS = 98.4 μS), which is sufficiently larger than original $G_{\mathrm{ref},2}$ value (viz., 0.35 μS). The Equation (2) is changed to account for the case where some odd-indexed cells in the as-fabricated very high resistance state are utilized. The original $G_{\mathrm{ref},1}$ of 1.25 μS is now altered to new conductance reference $G_{\mathrm{ref},5}$ (50 μS), which is approximately the midpoint conductance between the new low-resistance-state value (10 kΩ/ 100 μS) and high-resistance-state value (600 kΩ/ 1.6 μS). Thus, Equation (2) is modified to

$$b_{r_{2j+1}}^{(n)} = 1 \quad \text{if } C_{j+17}^{(n)} = 1 \ \& \ G_{2j+1} > 50\ \mu\text{S}, \qquad j = 0,\ 1,\ 2,\ \ldots,\ 15 \qquad (4)$$

$$= 0 \quad \text{otherwise}$$

The Equation (3) is kept the same since Equation (1), (4) now generates the same intermediate output bits with the new circuit logic for the final XOR operation in Equation (3). Utilizing the same 32-bit challenge with the write back process, these modifications ensure that the same final output bit is generated subsequently, which preserves the reliability of strong PUF.

Memristive cells that exhibit close conductance values might be susceptible to noisy conditions, resulting in decreased reliability of reading operations. In the reconfigured setting, as the cells disclose two conductance values that are slightly above/ below the $G_{\mathrm{ref},3}$ of ~3.33 μS, this could pose a problem. A slight fluctuation of resistance values near the threshold resistance could result in bit flips between ‘0’ and ‘1’. To mitigate this, we adopt the write back strategy utilized in literatures [6, 7]. In this work, the key is generated using stochasticity of the melt-

quenched high resistance state prior to the write back process, followed by storing the key in database. To ensure that the same key is generated by the end user, the conductance needs to be consistently above/ below reference conductance. This is to eliminate reading errors the next time the conductance value is read using same protocol. For example, in the case where one cell with a conductance of 3.57 μS and another cell with the conductance of 3.125 μS are disclosed, the write back process is performed by setting the cell with the lower conductance to the new high conductance of ~100 μS. Next, the reference conductance is increased to the approximate midpoint conductance (~50 μS) between the new high conductance value of ~100 μS and low conductance value of ~3.125 μS, so that the same protocol results in excellent reading operations with large sensing margin.

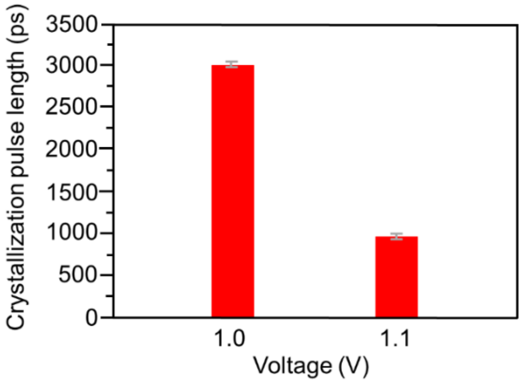

Figure. S9. Variation of crystallization pulse length of A PUF cells for different stimulus amplitudes. The range of values from experiments performed on three different cells in a batch is represented by the error bars.

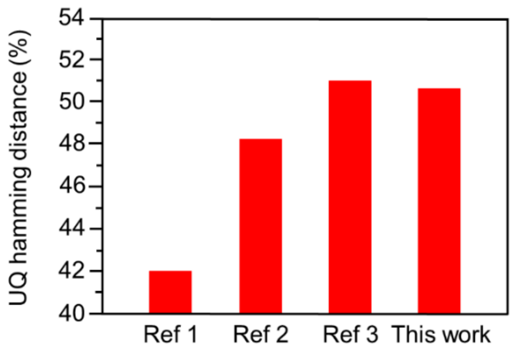

Figure S10. Comparison of mean value of HD distribution for UQ of weak A-PUF with that of state-of-the-art weak PUF.

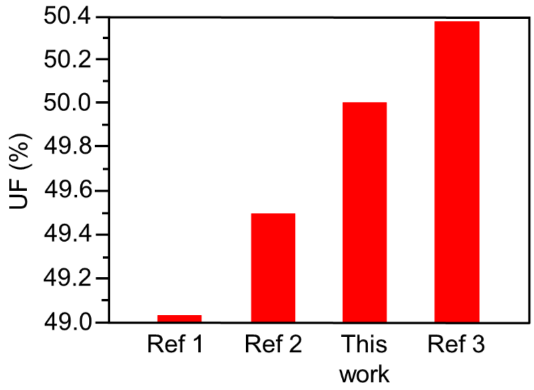

Figure S11. Comparison of mean value of HD distribution for UF of weak A-PUF with that of state-of-the-art weak PUF.

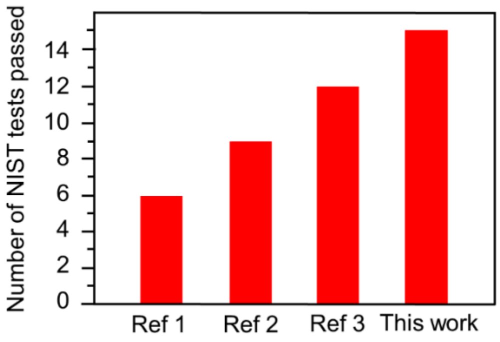

Figure S12. Comparison of number of NIST tests passed for strong A-PUF with that in other literatures.

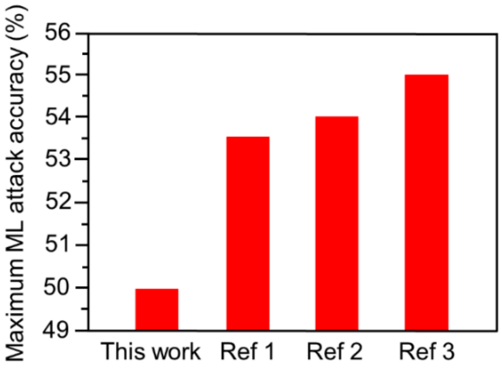

Figure S13. Comparison of mean value of maximum attack accuracy of strong A-PUF with that of state-of-the-art strong PUF.

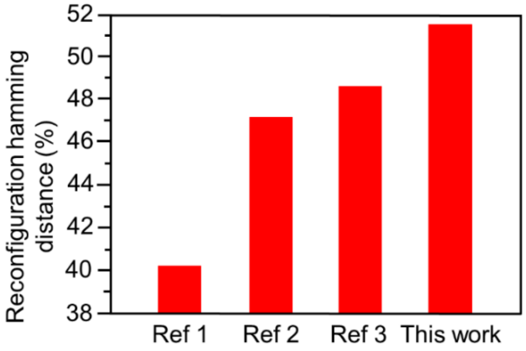

Figure S14. Comparison of mean value of HD distribution for reconfiguration of weak A-PUF with that of state-of-the-art weak PUF.

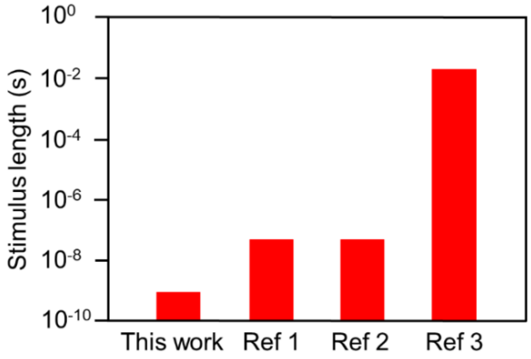

Figure S15. Comparison of stimulus length of A-PUF with that of state-of-the-art PUF.

References

[1] W. Che, J. Plusquellic, S. Bhunia, "A non-volatile memory based physically unclonable function without helper data", presented at *2014 IEEE/ACM International Conference on Computer-Aided Design (ICCAD)*, 2014.

[2] D. Ielmini, H.-S. P. Wong, *Nature electronics* **2018**, 1, 333.

[3] H. Jiang, C. Li, R. Zhang, P. Yan, P. Lin, Y. Li, J. J. Yang, D. Holcomb, Q. Xia, *Nature Electronics* **2018**, 1, 548.

[4] A. Chen, *IEEE Electron Device Letters* **2014**, 36, 138.

[5] L. Zhang, Z. H. Kong, C.-H. Chang, A. Cabrini, G. Torelli, *IEEE Transactions on Information Forensics and Security* **2014**, 9, 921.

[6] R. A. John, N. Shah, S. K. Vishwanath, S. E. Ng, B. Febriansyah, M. Jagadeeswararao, C.-H. Chang, A. Basu, N. Mathews, *Nature Communications* **2021**, 12, 1.

[7] J. Yang, X. Li, T. Wang, X. Xue, Z. Hong, Y. Wang, D. W. Zhang, H. Lu, "A physically unclonable function with BER< 0.35% for secure chip authentication using write speed variation of RRAM", presented at *2018 48th European Solid-State Device Research Conference (ESSDERC)*, 2018.
